# Associations of measured and genetically predicted leukocyte telomere length with vascular phenotypes: a population-based study

**DOI:** 10.1007/s11357-023-00914-2

**Published:** 2023-10-02

**Authors:** Dan Liu, N. Ahmad Aziz, Mohammed Aslam Imtiaz, Gökhan Pehlivan, Monique M. B. Breteler

**Affiliations:** 1 German Center for Neurodegenerative Diseases (DZNE), Population Health Sciences, Bonn, Germany; 2 Department of Neurology, Faculty of Medicine, University of Bonn, Bonn, Germany; 3 Institute for Medical Biometry, Informatics and Epidemiology (IMBIE), Faculty of Medicine, University of Bonn, Bonn, Germany

**Keywords:** Leukocyte telomere length, Polygenic risk score, Microvascular function, Cardiac function, Epigenome-wide association

## Abstract

Shorter leukocyte telomere length (LTL) is associated with cardiovascular dysfunction. Whether this association differs between measured and genetically predicted LTL is still unclear. Moreover, the molecular processes underlying the association remain largely unknown. We used baseline data of the Rhineland Study, an ongoing population-based cohort study in Bonn, Germany [56.2% women, age: 55.5 ± 14.0 years (range 30 – 95 years)]. We calculated genetically predicted LTL in 4180 participants and measured LTL in a subset of 1828 participants with qPCR. Using multivariable regression, we examined the association of measured and genetically predicted LTL, and the difference between measured and genetically predicted LTL (**Δ**LTL), with four vascular functional domains and the overall vascular health. Moreover, we performed epigenome-wide association studies of three LTL measures. Longer measured LTL was associated with better microvascular and cardiac function. Longer predicted LTL was associated with better cardiac function. Larger **Δ**LTL was associated with better microvascular and cardiac function and overall vascular health, independent of genetically predicted LTL. Several CpGs were associated (*p* < 1e-05) with measured LTL (*n* = 5), genetically predicted LTL (*n* = 8), and **Δ**LTL (*n* = 27). Genes whose methylation status was associated with **Δ**LTL were enriched in vascular endothelial signaling pathways and have been linked to environmental exposures, cardiovascular diseases, and mortality. Our findings suggest that non-genetic causes of LTL contribute to microvascular and cardiac function and overall vascular health, through an effect on the vascular endothelial signaling pathway. Interventions that counteract LTL may thus improve vascular function.

**Supplementary Information:**

The online version contains supplementary material available at 10.1007/s11357-023-00914-2.

## Introduction

Telomeres are repetitive DNA–protein structures, comprising thousands of tandem repeats of the TTAGGG sequence, located at the ends of chromosomes. They serve to maintain genomic stability and determine cellular lifespan [1, 2]. With each cell division, telomere length progressively shortens because of the inability of DNA polymerase to fully replicate the 3’ end of the DNA strand. When these sequences reach a critical length, the cellular DNA damage machinery is activated, which, in turn, triggers cellular senescence [1]. Telomere length is commonly measured as leukocyte telomere length (LTL), which is relatively easy to obtain from blood samples and is highly correlated with telomere length in other tissues [3]. LTL considerably varies across individuals [3–6], including across those of the same chronological age [7]. LTL has been proposed as a biomarker of biological aging as it reflects the amount of cellular turnover within an individual.

Many epidemiological studies have evaluated LTL in relation to a range of aging-related outcomes, including all-cause mortality [8–10], various types of cancer [11, 12], neurodegenerative diseases [13–16], chronic kidney diseases [17, 18], diabetes [19–21], cardiometabolic risk factors [22–24], and cardiovascular diseases (CVDs) [7–10, 19, 21, 25–27]. Although associations of shorter LTL with cardiovascular risk factors and CVDs have been repeatedly shown, observational studies investigating the association between LTL with preclinical vascular phenotypes and markers of vascular function, including endothelial function [28, 29], hemodynamics [30–36], arterial stiffness [37] and blood pressure traits [23, 24, 27, 34, 38–42] have yielded inconsistent results. Evidence from in vitro studies suggests that telomere shortening may be part of the mechanistic pathway leading to microvascular and hemodynamic dysfunction [4, 43, 44]. Aging and senescence induced by telomere shortening reportedly cause endothelial and hemodynamic dysfunction [45–47], and inhibition of telomere shortening has been shown to restore endothelial function and prevent the progression of atherosclerosis [48–50]. A detailed assessment of the relation between LTL and quantitative and sensitive preclinical vascular phenotypes, especially microvascular and hemodynamic function, could substantially advance our understanding of the role of telomere length in the pathogenesis of CVDs [4].

LTL has a strong inherited genetic component in humans, with an estimated heritability ranging from 44 to 86% [51, 52]. Recently, a large genome-wide association study (GWAS) found 197 independent sentinel variants associated with LTL, accounting for 4.54% of the variance in LTL [53]. Of note, these variants were located in gene regions involved in telomere regulation, maintenance, as well as cellular aging and senescence. Large-scale Mendelian Randomization studies have suggested a causal relationship between LTL and CVDs [2, 5, 26, 27, 53]. However, to what extent inherited genetic variation influencing LTL relates to markers of vascular function remains largely unknown.

Leveraging existing GWAS findings, we created weighted polygenic risk scores (PRSs) of LTL as proxies for genetically predicted LTL. This enabled us to investigate whether and to what extent measured and genetically predicted LTL, as well as the difference between the two measures, i.e. telomere length independent of genetic predisposition, are associated with vascular function phenotypes, including microvascular function, hemodynamics, arterial stiffness, and blood pressure, in the general population over a wide age range. Furthermore, we performed epigenome-wide association studies of the three LTL measures, followed by gene enrichment and pathway analyses, to gain biological insights into the mechanisms underlying the effects of LTL on vascular function.

## Methods

### Study population

Our research was based on cross-sectional baseline data from the Rhineland Study, an ongoing single-center, population-based cohort study among people aged 30 years and older in Bonn, Germany. All individuals from the age of 30 years onwards living in two pre-defined recruitment areas are invited to participate in the study. Participants are predominantly German of Caucasian descent. The only exclusion criterion is an insufficient command of the German language to provide informed consent. A primary objective of the Rhineland Study is to identify determinants and markers of healthy aging through a deep-phenotyping approach. At baseline, participants complete an 8-h in-depth multi-domain phenotypic assessment, and various types of biomaterials (including blood, urine, stool, and hair) are collected. Approval to undertake the study was obtained from the ethics committee of the University of Bonn, Medical Faculty. We obtained written informed consent from all participants in accordance with the Declaration of Helsinki.

Baseline data of the first 4180 participants of the Rhineland Study with both genetic data and vascular phenotype data were used to assess the association between genetically predicted LTL and vascular phenotypes. In a subset of participants, both measured LTL and vascular phenotypes were available (*n* = 1828).

### Measurement of leukocyte telomere length

LTL was manually measured using the quantitative polymerase chain reaction (qPCR) method adapted from the previously published original method [54]. Blood samples were collected between 7:00 to 9:45 in the morning from an antecubital or dorsal hand vein. Genomic DNA was extracted from buffy coat fractions of anti-coagulated blood samples using Chemagic DNA buffy coat kit (PerkinElmer, Germany) and stored at -80 °C before use. LTL was measured as the relative quantities (T/S ratio) of the telomeric TTAGGG repeat (T) and the single copy of a housekeeping gene, albumin (S). Each reaction contained 25 ng of DNA, 400 nM of the telomere length primers (tel-forward: ACA CTA AGG TTT GGG TTT GGG TTT GGG TTT GGG TTA GTGT; tel-reverse: TGT TAG GTA TCC CTA TCC CTA TCC CTA TCC CTA TCC CTA ACA) and 200 nM of the albumin primers (alb-forward: CGG CGG CGG GCG GCG CGG GCT GGG CGG AAA TGC TGC ACA GAA TCC TTG; alb-reverse: GCC CGG CCC GCC GCG CCC GTC CCG CCG GAA AAG CAT GGT CGC CTG TT) and 1 × SYBR Green PCR Mastermix (iTaq Universal SYBR Green Supermix). Seven concentrations of a reference DNA sample spanning a 128-fold range of DNA concentration (i.e. from 230 ng/ μl to 3.59 ng/ μl in twofold dilution steps) as well as the negative controls, were included in every run. The reactions were performed in triplicates for each sample using the 7900HT machine (Applied Biosystems). At the end of cycling, a dissociation curve was included to detect abnormal PCR products. A standard curve of each primer was assessed for quality control. Primers achieving 90–110% reaction efficiency and an R^2^ across the linear range > 0.99 were considered acceptable. No amplification in negative controls was acceptable. The Ct (cycle threshold) values that had a coefficient of variance of more than 1% of each sample were excluded from further analysis. The resulting T/S ratio was calculated for each well and the mean value of the triplicates was reported.

Based on DNA methylation levels, the relative proportion of twelve leukocyte subtypes, including basophils, eosinophils, neutrophils, monocytes, naïve B cells, memory B cells, naïve CD4T cells, memory CD4T cells, regulatory T cells, naïve CD8T cells, memory CD8T cells and natural killer cells, was derived using the “FlowSorted.BloodExtended.EPIC” R package, which is based on a reference-based deconvolution method described by Salas and colleagues [55]. To investigate the potential effects of cell type compositions of leukocytes on LTL, we further assessed the correlation between measured LTL and the twelve leukocyte subtypes.

### Genetically predicted leukocyte telomere length

DNA was extracted from buffy coat samples and genotyped using Infinium Omni2.5Exome-8 BeadChip containing 2,612,357 SNPs and processed using GenomeStudio (version 2.0.5). Quality control of genotypes was performed using PLINK (version 1.9). Single-nucleotide polymorphisms (SNPs) exclusion criteria were Hardy–Weinberg disequilibrium (*p* < 1*10^–6^), minor allele frequency (< 0.01) and poor genotyping rate (< 99%) [56]. Samples with poor call rate (< 95%), abnormal heterozygosity, cryptic relatedness and gender mismatch were excluded. Since variation in population structure can cause systematic differences in allele frequencies, we used EIGENSTRAT (version 16000), which uses principal components (PCs) to detect and correct for variation in population structure [57]. Based on the EIGENSTRAT estimation, we excluded cases of non-Caucasian descent, retaining only participants from Caucasian descent for analysis. We used the 1000 Genomes phase 3 reference panel for the imputation of missing genotypes using impute2 (version 2). To include only SNPs with high imputation quality, we filtered the SNPs based on an info score metric > 0.3 [58].

Genetically predicted leukocyte telomere length was calculated based on two GWASs. Our primary analyses were based on the GWAS by Codd et al., which included 472,174 UK Biobank participants and identified 197 variants associated with LTL at genome-wide significance (5*10^–8^) [53]. Importantly, these variants were located in gene regions with known roles in 1) telomere regulation: genes encoding components of the telomere SHELTRIN complexes, alternative lengthening of telomeres pathway and factors that post-translationally modify key telomere proteins; 2) telomerase regulation: genes encoding core components of proteins that regulate the assembly and activity of telomerase and genes involved in TERC stability, intracellular trafficking and processing which is important before telomerase assembly; 3) telomere maintenance: DNA replication, recombination, and repair. Therefore, these variants are likely to be causally associated with LTL [53]. Moreover, the 130 SNPs selected by Codd et al. for their Mendelian Randomization study, which we used for calculating PRS_MRCodd_, were non-pleiotropic. To remove potentially pleiotropic loci, Codd et al. investigated the identified variants for association with 558 traits using previously curated data [59]. For each variant, evidence of pleiotropy was defined as associations within at least three different domains, which led to the selection of 130 conditionally independent, uncorrelated, and non-pleiotropic genome-wide significant instruments [53]. Thus, the identified associations between genetically predicted LTL and vascular function are likely through only genetically determined telomere length. We calculated two weighted PRS for LTL: PRS_GWSCodd_ included 150 variants that reached genome-wide significance, and PRS_MRCodd_ included the MR instrument variants. Individual SNPs were coded for effect allele dosage associated with longer LTL, ranging from zero (no effect alleles) to two (two effect alleles). The published regression coefficient (beta) estimates representing the per-allele effect on normalized LTL were assigned as weights for each SNP (Table S1**)**. As a sensitivity analysis, we also created PRSs based on a GWAS by Li et al., which included 78,592 individuals of European descent [5]. They identified 52 variants independently associated with LTL at a false discovery rate (FDR) < 0.05. Among these, 20 sentinel variants reached genome-wide significance (5*10^–8^); 47 out of the 52 identified SNPs were available in our genetic array. PRS_FDRLi_ was calculated using 47 variants that were significant at FDR < 0.05 and PRS_GWSLi_ was calculated using the 20 sentinel variants that reached genome-wide significance. These four PRSs were further standardized to have a mean of 0 and a standard deviation of 1 and were used in the analyses as proxies for genetically predicted LTL.

### Difference between measured and genetically predicted LTL

We defined delta LTL (**Δ**LTL: **Δ**LTL_MRCodd_, **Δ**LTL_GWSCodd_, **Δ**LTL_FDRLi_, **Δ**LTL_GWSLi_) as the difference between measured and genetically predicted LTL for each participant, and estimated it as the residual remaining after regressing measured LTL on PRS of LTL, adjusting for batch information of measured LTL and the first 10 genetic PCs.

### Assessment of microvascular function

Microvascular function was assessed as reactive skin hyperemia (RSH) with a laser Doppler flowmetry device (Moors, UK) using a local thermal heating protocol. Skin blood flow (SBF) was measured on the ventral surface of the forearm for a total of 26 min. After 2 min of baseline SBF measurement, the area of interest was heated up to 40 degrees Celsius with an integrated heating probe, and the temperature was kept constant until the end of the examination. The peak in baseline SBF is followed by a nadir and after approximately 20 min it reaches a plateau, which is associated with the nitric oxide production capacity of the endothelial cells [60]. RSH was calculated as the percentage increase in SBF from baseline to the last 2 min of plateau level ([(Plateau SBF—Baseline SBF) / Baseline SBF)] × 100).

### Assessment of hemodynamic parameters

Hemodynamics was quantified as cardiac index (CI, L/min/m^2^), systemic vascular resistance index (SVRI, dynes/sec/cm^5^/m^2^) and stroke index (SI, mL/m^2^). Cardiovascular examinations were performed in temperature-controlled rooms after the acclimatization of the participants in the study centers. Pulsatile, resistive and flow-related hemodynamics were obtained beat-to-beat in the supine position after 5 min of rest with an impedance cardiography device, which registers simultaneously electrocardiography (ECG) signals and measures blood pressure at 2-min intervals. Briefly, cardiac output (CO, L/min) was computed as stroke volume (SV, mL) multiplied by heart rate (beat per minute). CI was computed as CO divided by body surface area (BSA, m^2^). SVRI was calculated as mean arterial pressure (MAP, mmHg) divided by CO, multiplied by 80. SI was computed as SV divided by BSA.

### Assessment of arterial stiffness

Arterial stiffness was quantified as total arterial compliance index (TACI, mL/mmHg/m^2^), aorta-femoral pulse wave velocity (PWV, m/s) and Ankle-Brachial Index (ABI). TACI was determined as dividing SV with brachial pulse pressure (PP, mmHg) and BSA. An integrated oscillometric femoral blood pressure cuff was used to determine aorta-femoral pulse wave velocity (PWV, m/s). The propagation time of the pulse wave was estimated as the delay between the opening of the aortic valve determined with impedance cardiography (ICG) waves and the arrival of the pulse wave to the mid-femoral cuff. PWV was calculated as the distance measured between the supra-sternal notch and the mid-femoral cuff divided by propagation time. Ankle-Brachial Index (ABI) was approximated as systolic blood pressure (SBP, mmHg) measured at the ankle divided by SBP measured at the upper arm on the same body-side, and measured separately for the left and right body sides. The lower value of ABI was used in the analyses in cases where ABI was lower than 1.40, otherwise, the higher value was used, as recommended previously [61].

### Assessment of blood pressure

Systolic blood pressure (SBP, mmHg) and diastolic blood pressure (DBP, mmHg) were measured three times (separated by ten minutes intervals), using an oscillometric blood pressure device (Omron 705 IT). The measurements were performed while people were sitting in a resting chair in a quiet environment, and the average of the second and third measurements was used for further calculation. Mean arterial pressure (MAP) was calculated as (SBP + 2 × DBP)/3. Pulse pressure (PP) was defined as the difference between SBP and DBP.

### Assessment of overall vascular health

An overall vascular health index was calculated as an average Z-score based on nine vascular phenotypes, including reactive skin hyperemia, cardiac index, systemic vascular resistance index, stroke index, total arterial compliance index, pulse wave velocity, ankle-brachial index, mean arterial pressure and pulse pressure.

### Demographic and health variables

We included age, sex, and education level as demographic covariates. Education level was grouped as less than high school, high school, or higher. Smoking status was defined as current smokers or non-current smokers. Body mass index (BMI) was calculated as weight in kilograms divided by height in meters squared.

### Statistical analysis

Data were summarized as mean ± standard deviation (SD) or counts with proportions, for continuous and categorical variables, respectively. All vascular phenotypes and LTL measures were standardized using z-scores before further analyses to enable a better comparison of the effect sizes across different physiological domains.

### Association of measured LTL, genetically predicted LTL, ΔLTL with vascular phenotypes

We used multiple linear regression analyses to assess the association between measured LTL and each vascular phenotype. Models were adjusted for age, sex (women versus men), batch information of LTL, smoking status (current smokers versus non-current smokers) and BMI (model: vascular phenotype ~ measured LTL + age + sex + batch information of LTL + BMI + smoking status). As a previous study found that controlling for leukocyte compositions attenuated the association between LTL and cardiovascular risk factors by between 10 to 20% [23], we further adjusted for cell type proportions estimated from the same DNA samples, using a previously described method [55], as a sensitivity analysis.

To assess the association between measured and genetically predicted LTL, we first evaluated whether the previously reported genetic variants of LTL were associated with measured LTL in our cohort. Next, we examined the association between PRS and measured LTL. Third, we assessed the association between PRS and each vascular phenotype. All analyses were performed using multiple linear regression, adjusting for age, sex (women versus men), the first 10 genetic principal components (PCs) to account for population stratification, smoking status (current smokers versus non-current smokers) and BMI (model: vascular phenotype ~ PRS of LTL + age + sex + first 10 genetic PCs + BMI + smoking status). Finally, the association between **Δ**LTL and each vascular phenotype was assessed while adjusting for age, sex (women versus men), PRS, smoking status (current smokers versus non-current smokers) and BMI (model: vascular phenotype ~ ΔLTL + age + sex + BMI + smoking status + PRS of LTL). Our primary analyses were based on PRS_GWSCodd_ and PRS_MRCodd_. As sensitivity analyses, we also perform the analyses with PRS_FDRLi_ and PRS_GWSLi_.

To assess whether age and sex modified the associations between LTL and vascular phenotypes, we added interaction terms for the interaction between age and sex with vascular phenotype to the regression models. In case of significant interaction effects, additional sex-stratified analyses were performed.

All statistical analyses were performed using R version 3.5.2. All standardized effect estimates are reported with their 95% confidence intervals (CIs). We had very specific a priori hypotheses regarding the associations of telomere length with microvascular and hemodynamic function based on the literature [4, 43–47], therefore, we did not correct for multiple testing for the association between LTL measures and vascular phenotypes and set the level of statistical significance at *P* < 0.05.

### Epigenome-wide association study of LTL measures and gene enrichment analyses

We investigated the associations between LTL measures (independent variable) and DNA methylation level (dependent variable) using multiple linear regression while adjusting for age, sex, batch effects, blood cell proportion, and the first 10 genetic PCs. FDR adjustment was applied to account for multiple comparisons: FDR adjusted q < 0.05 was considered epigenome-wide significant, while *p* < 1e-05 was considered to indicate suggestive significance.

Cytosine-phosphate-Guaniene sites (CpGs) showing associations with LTL measures at *p* < 1e-05 were searched in EWAS Catalog [62] and EWAS Atlas [63] to identify associated traits reported in previous EWAS. We also searched the known associations of the mapped gene for each CpG in previously published GWAS using the GWAS catalog [64]. We conducted further gene enrichment analyses using Gorilla [65] based on a significance-ranked gene list (i.e. from the lowest to the highest *p*-value of the corresponding CpG site) and summarized the results using the REViGO tool [66]. We also conducted Kyoto Encyclopedia of Genes and Genomes (KEGG) pathway analysis using the R missMethyl package [67].

## Results

We included 4180 participants (56.2% women) with data on PRS of LTL and vascular phenotypes, with a mean age of 55.5 years (SD = 14.0 years, range 30 – 95 years). In the subset of 1828 participants with additionally measured LTL data, the mean age was 54.8 years (SD = 14.1 years, range from 30 – 95 years), and 56.8% were women. The two datasets did not statistically significantly differ regarding age, sex and BMI distribution. A summary of the characteristics of the study population is provided in Table 1.

**Table 1:** Characteristics of the study population

	Participants with genetically predicted LTL data (*n* = 4180)	Participants with measured LTL data^#^ (*n* = 1828)	Adjusted *p*-value*
Age, year			
Mean (SD)	55.5 (14.0)	54.8 (14.1)	0.10
Median [Min, Max]	55.0 [30.0, 95.0]	54.0 [30.0, 95.0]	
Sex, n (%)			0.32
Women	2349 (56.2%)	1038 (56.8%)	
Men	1831 (43.8%)	790 (43.2%)	
Body mass index, kg/m^2^, mean (SD)	25.8 (4.4)	25.8 (4.6)	0.50
Current smoking, n (%)	512 (12.2%)	257 (14.1%)	0.05
Measured LTL, mean (SD)	-	1.0 (0.3)	-
Vascular phenotypes, mean (SD)			
Reactive skin hyperemia	499 (492)	485 (447)	0.06
Cardiac index, L/min/m^2^	3.2 (0.5)	3.2 (0.5)	0.64
Systemic vascular resistance index, dynes· sec/cm^5^/m^2^	2120 (469)	2120 (475)	0.13
Stroke index, mL/m^2^	52.1 (8.7)	51.7 (8.5)	0.01
Total arterial compliance index, mL/mmHg/m^2^	1.1 (0.3)	1.0 (0.3)	0.01
Pulse wave velocity, m/s	6.8 (2.9)	6.9 (3.6)	0.12
Ankle-Brachial index	1.2 (0.1)	1.1 (0.1)	0.25
Systolic blood pressure, mmHg	127 (16.1)	128 (16.5)	< 0.01
Diastolic blood pressure, mmHg	75.4 (9.3)	76.9 (9.6)	< 0.01
Mean arterial pressure, mmHg	92.6 (10.6)	93.9 (10.9)	< 0.01
Pulse pressure, mmHg	51.6 (10.5)	51.7 (10.7)	0.14

*LTL* leucocyte telomere length, *SD* standard deviation

^#^ Participants with measured telomere length data (*n* = 1828) is a subset from 4180 participants with genetically predicted LTL data

^*^ Comparison between two datasets, adjusted for age and sex

Measured LTL was strongly associated with chronological age: measured LTL decreased 0.006 SD per year [95% CI: (-0.007, -0.005), *p*-value < 2e-1]. Compared to men, women’s measured LTL were 0.02 SD longer [95% CI: (0.05, 0.01), *p*-value = 0.04] (Figure S1). Measured LTL was weakly correlated with leukocyte subtypes (all r < 0.23, Figure S2).

### The association between measured LTL and vascular phenotypes

**Fig. 1:**
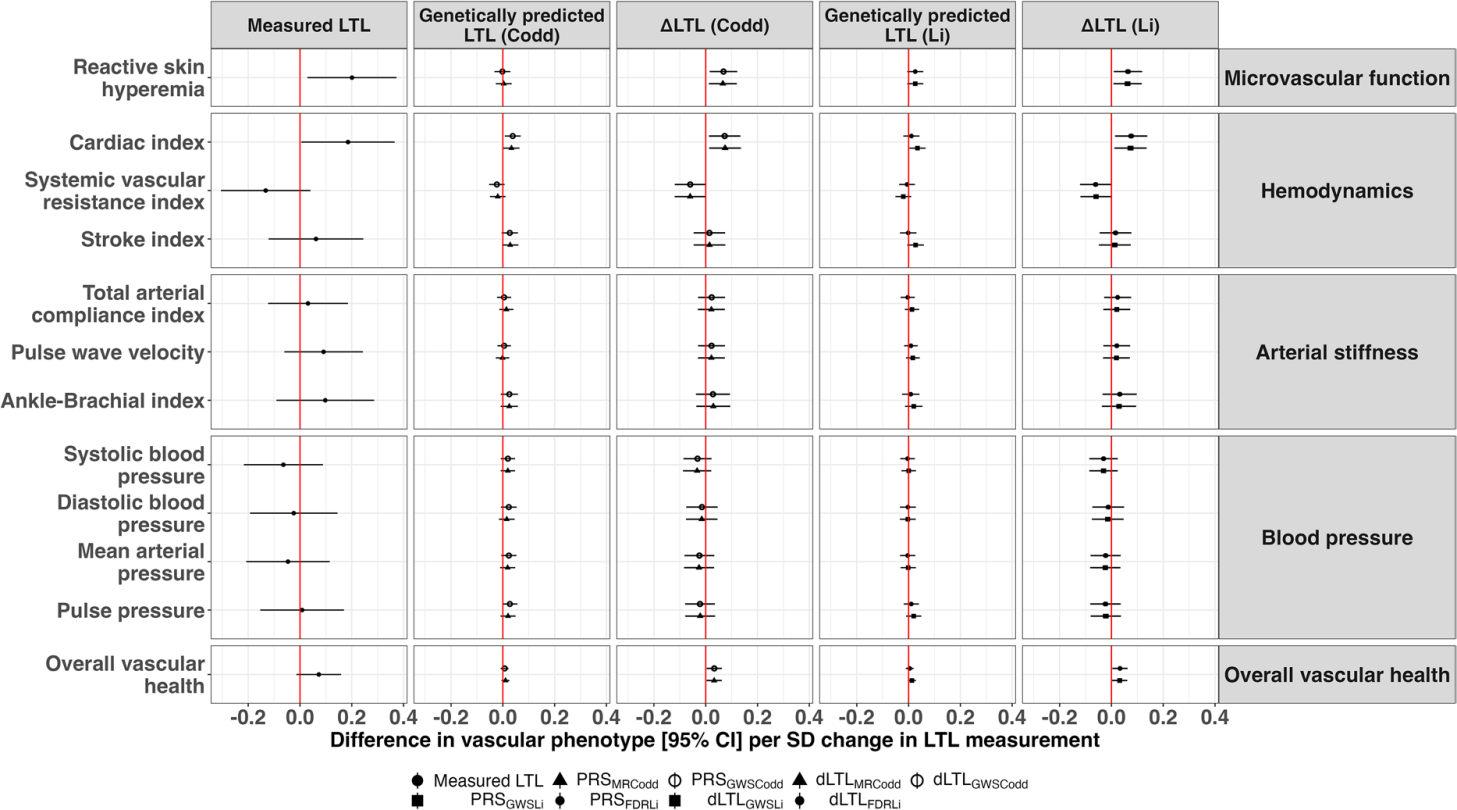
The association between leukocyte telomere length measurements and vascular phenotypes. Abbreviation: LTL, leukocyte telomere length; ΔLTL, difference between measured LTL and genetically predicted LTL; PRS, polygenetic risk score; FDR, false discovery rate; GWAS, genome-wide association study; CI, confidence interval; SD, standard deviation. PRS_GWSCodd_ includes 150 variants at the genome-wide significance level (5*10^–8^) and PRS_MRCodd_ includes 100 conditionally independent, uncorrelated and non-pleiotropic variants used as instruments for Mendelian Randomization (MR) in a previously study by Codd et al. [53]. PRS_FDRLi_ includes 47 variants at false-discovery rate < 0.05 and PRS_GWSLi_ of LTL includes 20 variants at genome-wide significance level (5*10^–8^). Models for each LTL measurement: Vascular phenotype ~ measured LTL + age + sex + batch information of LTL + body mass index + smoking status; Vascular phenotype ~ PRS of LTL (PRS_MRCodd_/PRS_GWSCodd_/ PRS_FDRLi_/ PRS_GWSLi_) + age + sex + first 10 genetic principal components + body mass index + smoking status; Vascular phenotype ~ **Δ**LTL (dLTL_MRCodd_/ dLTL_GWSCodd_/dLTL_FDRLi_/dLTL_GWSLi_) + age + sex + body mass index + smoking status + PRS of LTL

Longer measured LTL was associated with better microvascular function and cardiac index: each SD increase in measured LTL was associated with 0.20 SD (95% CI: 0.03, 0.37) increase in reactive skin hyperemia, and 0.19 SD (95% CI: 0.01, 0.37) increase in cardiac index. Longer measured LTL was also associated with a lower systemic vascular resistance index, although this association did not reach statistical significance [-0.13 SD change (95% CI: -0.30, 0.04)]. Measured LTL was not significantly associated with arterial stiffness or blood pressure phenotypes (Fig. 1). The associations between measured LTL and microvascular and cardiac function remained significant after further adjustment of cell type proportions.

### The associations of the previously reported variants and PRS of LTL with measured LTL

When evaluating individual SNPs that had previously been associated with LTL by Codd et. al [53], we could replicate the genes (loci) with established roles in telomere biology: TERC (rs2293607) and TERT (rs2853677, rs138895564, rs79717857) regulate the formation and activity of telomerase; STN1/OBFC (rs9419958, rs10748858), RTEL1 (rs2259797, rs8114049, rs187577818) and CTC1 (rs75664430) regulate the telomere structure; SAMHD1 (rs6030416) and TYMS (rs111811424) regulate the nucleotide metabolism (Table S1a). Regarding other variants, although the *p*-values did not reach statistical significance, the direction of the associations and the magnitude of the estimates were quite similar to those reported previously. The replication results on prior GWAS by Li et al. [5] were quite similar: we replicated the previously reported top variants, including rs10936600 (*TERC*), rs2853677 (*TERT*), rs9419958 (*OBFC1*), rs75691080 (*STMN3*), with the same direction and even larger effect sizes (Table S1b).

All PRSs were associated with longer measured LTL in our cohort: each SD increase in PRSs was associated with around 0.14 SD increase (95% CI: 0.10, 0.19) in measured LTL. Taken together, these findings support the reliability of our LTL measurements and further validate the derived genetic instruments.

### The association between genetically predicted LTL and vascular phenotypes

Longer genetically predicted LTL was associated with a better cardiac function (0.04 SD increase (95% CI: 0.01, 0.07) in cardiac index per SD increase in PRS_GWSCodd_ and PRS_MRCodd_). In the sensitivity analysis, an increase in PRS_GWSLi,_ but not PRS_FDRLi,_ was associated with a better cardiac index, indicating that the PRS calculated using the genome-wide significant sentinel variants associated with LTL was informative and included less noise. There was no significant association of genetically predicted LTL with arterial stiffness traits and blood pressure (Fig. 1). The associations between genetically predicted LTL and vascular phenotypes were virtually identical in the subset of 1828 participants with measured LTL data.

To explore which variants drove the associations between PRS and cardiac index, we further investigated the associations of the individual genetic variants with cardiac index. Alleles mapping to distinct genes, including *CDA, SLC16A4, ACYP2, LINC01122, SMC4*, *POT1 and STN1 (OBFC1),* were associated with cardiac index (Figure S3). These genes are involved in DNA damage repair and nucleotide metabolism, which both play a vital role in cellular senescence [2, 5].

### The association between ΔLTL and vascular phenotypes

Longer measured than genetically predicted LTL (higher **Δ**LTL) was associated with better microvascular and cardiac function independent of genetically predicted LTL: each SD increase in **Δ**LTL was associated with 0.07 SD increase (95% CI: 0.02, 0.12) in reactive skin hyperemia, 0.08 SD increase (95% CI: 0.02, 0.13) in cardiac index, and 0.06 SD decrease (95% CI: -0.12, -0.01) in systemic vascular resistance index. **Δ**LTL was not associated with arterial stiffness and blood pressure traits. Age and sex did not significantly modify the association of LTL measures with endothelial function and cardiac index (all interaction *P* values > 0.10). Overall, both the magnitudes and directions of the associations with vascular phenotypes were consistent in measured, genetically predicted LTL and **Δ**LTL (Fig. 1).

In addition, we found that ΔLTL was associated with the overall vascular health index (Fig. 1). However, measured LTL and genetically predicted LTL were not associated with the overall vascular health index, which indicates that primarily the non-genetically determined LTL contributes to overall vascular health.

### EWAS of LTL measures and gene enrichment analyses

**Fig. 2:**
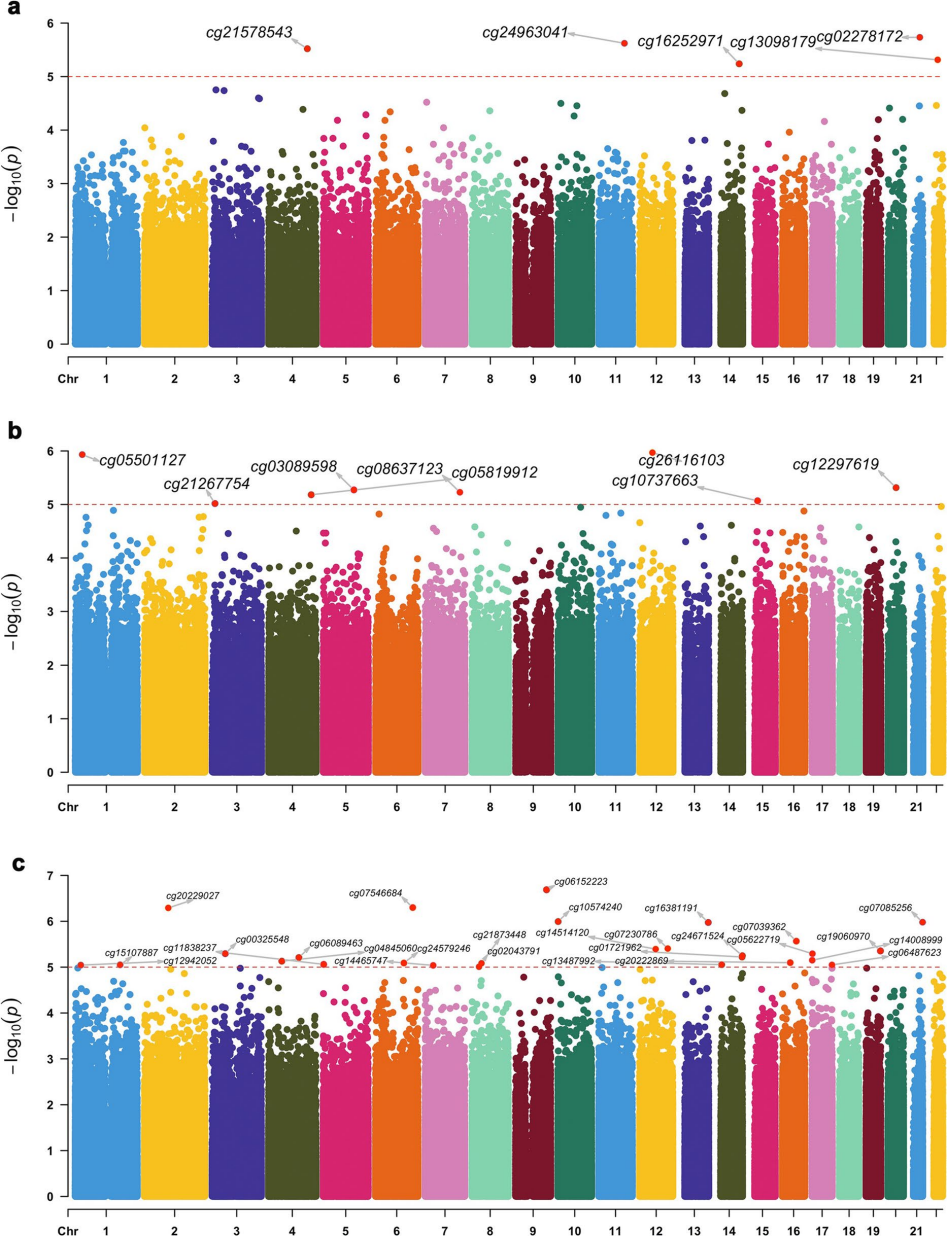
Epigenome-wide association results of leukocyte telomere length measurements. Manhattan plots of the epigenome-wide association study (EWAS) results for (**a**) measured leukocyte telomere length and (**b**) genetically predicted leukocyte telomere length and (**c**) delta leukocyte telomere length. The x-axis depicts sites ordered by chromosomal position with the respective -log_10_ *p*-value on the y-axis. The red dash horizontal line represents the level of significance at *p*-value < 1e-05

We identified 5 CpGs associated with measured LTL (Fig. 2a), 8 CpGs associated with genetically predicted LTL (Fig. 2b), and 27 CpGs associated with **Δ**LTL (Fig. 2c) at *p* < 1e-05 level.

The CpGs we found associated with measured LTL have previously been linked to cardiac function (i.e. electrocardiogram morphology, QT interval, QT dynamics), and the CpGs associated with genetically predicted LTL have been linked to blood pressure traits (Table 2a and 2b). The CpGs associated with **Δ**LTL have been linked to environmental exposure (i.e. air pollution, nitrogen dioxide exposure, HIV infection), lifestyle factors (i.e. alcohol consumption, vitamin B12 supplement) and mortality in previous EWAS. Moreover, the mapped genes have been previously associated with CVDs (i.e. coronary artery disease, myocardial infarction, ischemic stroke) and other aging-related phenotypes (i.e. cancer, chronic kidney disease, type 2 diabetes, Alzheimer’s diseases, hand grip strength, cognition, neurofibrillary tangles, and cortical thickness) (Table 2c).

**Table 2:** CpG sites associated with leukocyte telomere length measurements and other EWAS and GWAS traits

a. CpG sites associated with measured LTL at a suggestive level (1e-05)										
CpG	chr	pos	Nearest Gene	Relation to Island	beta	Standard error	*P*-value	fdr	Other EWAS trait association	Other GWAS trait association
cg02278172	chr21	35,828,165	KCNE1	N_Shelf	-1.05e-02	2.19e-03	1.85e-06	6.69e-01		Electrocardiogram morphology (amplitude at temporal datapoints), Electrocardiographic traits (multivariate), QT interval, QT dynamics during exercise, QT dynamics during recovery from exercise, QTc interval, Metabolite levels, L-selectin levels, Bilateral cleft lip and palate, Educational attainment, Long QT syndrome
cg13098179	chr22	30,789,374	OSBP2	N_Shelf	-8.19e-03	1.79e-03	4.86e-06	1.00e + 00	atopy, LILRB3 protein levels	Protein quantitative trait loci (liver), Insomnia, Self-reported math ability (MTAG), Self-reported math ability, Highest math class taken (MTAG), Colorectal cancer, Takayasu arteritis, Educational attainment (years of education), Educational attainment (MTAG), Red cell distribution width, Mean reticulocyte volume, Sulfatide (d18:1/16:0) levels, Total Sulfatide levels, Lifetime smoking index, Response to ketamine in bipolar disorder or major depression (antidepressant effects), Skin pigmentation, Adverse response to chemotherapy (neutropenia/leucopenia) (gemcitabine), interferon-related traits, Constipation, Menarche (age at onset), Estimated glomerular filtration rate change in renal transplantation (donor effect), Early-onset schizophrenia, Educational attainment, Neurofibrillary tangles (SNP x SNP interaction), Intelligence
cg16252971	chr14	93,125,602	RIN3	OpenSea	-7.52e-03	1.65e-03	5.77e-06	1.00e + 00	atopy, eosinophilia	Post bronchodilator FEV1/FVC ratio, Eosinophil counts, Hip index, Monocyte count, Tenofovir clearance in HIV infection, Chronic obstructive pulmonary disease, Chronic obstructive pulmonary disease (moderate to severe), Chronic obstructive pulmonary disease (severe), Neutrophil count, Total body bone mineral density (age 0–15), Bone mineral density (paediatric, total body less head), Bone mineral density (paediatric, lower limb), White blood cell count, Estimated glomerular filtration rate, Estimated glomerular filtration rate (creatinine), Appendicular lean mass, Breast cancer, Lung function (FVC), Myeloid white cell count, White blood cell count, Lymphocyte percentage of white cells, Mean platelet volume, Lung function (FEV1), FEV1, Subcortical volume (MOSTest), Subcortical volume (min-P), Plateletcrit, Serum alkaline phosphatase levels, Pulse pressure, Platelet distribution width, Primary biliary cholangitis, Whole brain restricted isotropic diffusion (multivariate analysis), Vertex-wise sulcal depth, Local histogram emphysema pattern, Asthma, Basophil count, Systemic juvenile idiopathic arthritis, Brain morphology (min-P), Sum basophil neutrophil counts, Sum eosinophil basophil counts, Post bronchodilator FEV1, Breast cancer (estrogen-receptor negative), Sum neutrophil eosinophil counts, Granulocyte count, Monokine induced by gamma interferon levels, Composite immunoglobulin trait (IgA/IgM), Chronic obstructive pulmonary disease or resting heart rate (pleiotropy), Neutrophil percentage of white cells, Metabolic biomarkers (multivariate analysis), Cortical surface area, Rheumatoid arthritis, Serum albumin levels, Eczema, Paget's disease, Sepsis (28-day mortality), TB-LM or TBLH-BMD (pleiotropy), Blood protein levels, Proximal colorectal cancer, Eosinophil percentage of white cells, Cortical thickness, Body fat percentage, Hair color, Dialysis-related mortality, Childhood ALL/LBL (acute lymphoblastic leukemia/lymphoblastic lymphoma) treatment-related venous thromboembolism, Core binding factor acute myeloid leukemia
cg21578543	chr4	155,312,795	DCHS2	OpenSea	-8.92e-03	1.90e-03	3.02e-06	8.22e-01	Tissue, age	Facial morphology (nose height), Facial morphology (nose roundness 1), Facial morphology (nose roundness 3), Facial morphology (columella inclination), Facial morphology (nostril size), Facial morphology (segment 27), Facial morphology, Facial morphology traits (63 three-dimensional facial segments), facial morphology traits (multivariate analysis), Facial morphology (segment 2), Serum levels of protein ALDH1A3, Serum levels of protein SYK, Blood protein levels, Schizophrenia, bipolar disorder or major depressive disorder, Schizophrenia, bipolar disorder or recurrent major depressive disorder, Cortical surface area, Vertex-wise cortical surface area, Fibrinogen, Food allergy (maternal genetic effects), Bone mineral density (spine), Velopharyngeal dysfunction, Fatty acid-binding protein, adipocyte levels, radiation-induced toxicity (physician-rated acute xerostomia), Schizophrenia, bipolar disorder or recurrent major depressive disorder x sex interaction, UBA737 abundance in stool, Blond vs. brown/black hair color, Apolipoprotein B levels, Vertex-wise sulcal depth, Ischemic stroke, Venous thromboembolism or fibrinogen levels (pleiotropy), Sib-shared facial trait 522; Facial segment 17; 3D morphology of the upper lip, Coronary artery disease or fibrinogen levels (pleiotropy), Total cholesterol levels, C-reactive protein, Low density lipoprotein cholesterol levels, Type 2 diabetes (age of onset), Folding of antihelix, Immune reponse to smallpox (secreted IL-1beta), Bilirubin levels, Alzheimer's disease (age of onset), Thyrotoxic hypokalemic periodic paralysis and Graves disease, Venous thromboembolism, Atopic dermatitis (moderate to severe), Hair color
cg24963041	chr11	102,402,413	MMP7	OpenSea	-6.52e-03	1.38e-03	2.39e-06	6.89e-01	age	Matrix metalloproteinase-7 levels, MMP 7 plasma levels, Blood protein levels, Prostate cancer, Serum levels of protein MMP7, Prostate-specific antigen levels, TestASV_19 (Prevotella) prevalence, Chronotype (sMEQ score), Cardio-cerebrovascular disease in dyslipidemia, Neuroblastoma, Matrilysin levels, Narcolepsy with cataplexy
b. CpG sites associated with genetically predicted LTL at a suggestive level (1e-05)										
CpG	chr	pos	Nearest Gene	Relation to Island	beta	Standard error	*P*-value	fdr	Other EWAS trait association	Other GWAS trait association
cg26116103	chr12	51,157,776	ATF1	Island	-9.90e-01	2.03e-01	1.07e-06	4.25e-01	age, Tissue	Sex hormone-binding globulin levels, Sex hormone-binding globulin levels adjusted for BMI, Sex hormone-binding globulin levels in postmenopausal women, Sex hormone-binding globulin levels in premenopausal women, Mean corpuscular volume, Mean spheric corpuscular volume, Liver enzyme levels (alanine transaminase), Alanine aminotransferase levels, Estimated glomerular filtration rate, Systolic blood pressure, HDL cholesterol levels, Educational attainment (years of education), Educational attainment (MTAG), Colorectal cancer, Apolipoprotein A1 levels, Varicose veins, Diastolic blood pressure, Male-pattern baldness, Multiple sclerosis, Subcortical volume (MOSTest), Male-pattern baldness, Blood glucose levels, Mean arterial pressure, Colorectal cancer or advanced adenoma, Sudden cardiac arrest, Ascending aorta minimum area, Colon polyp, Spleen volume
cg05501127	chr1	27,732,648	WASF2	OpenSea	2.80e + 00	5.75e-01	1.17e-06	4.61e-01		Systolic blood pressure, Medication use (calcium channel blockers), Pulse pressure, Monocyte count, Serum levels of protein MASP1, Estradiol plasma levels (breast cancer)
cg12297619	chr20	32,308,323	PXMP4	S_Shore	-1.01e + 00	2.22e-01	4.85e-06	7.30e-01	age, Triglycerides to total lipids ratio in medium VLDL	Sex hormone-binding globulin levels, Sex hormone-binding globulin levels adjusted for BMI, Sex hormone-binding globulin levels in postmenopausal women, Height, Heel bone mineral density, Appendicular lean mass, Diastolic blood pressure, Post bronchodilator FEV1/FVC ratio, Protein quantitative trait loci (liver), Estimated glomerular filtration rate (creatinine), Alanine aminotransferase levels, Waist circumference adjusted for body mass index, Mean corpuscular hemoglobin, Glycated hemoglobin levels, Triglyceride levels, Platelet count, Abdominal fat cell number, Aspartate aminotransferase to alanine aminotransferase ratio
cg03089598	chr5	122,544,575	SNCAIP	OpenSea	1.82e + 00	4.00e-01	5.34e-06	7.30e-01		Vertical cup-disc ratio, Brain shape (segment 1), Whole brain free water diffusion (multivariate analysis), Cortical surface area, Vertex-wise cortical surface area, Cortical thickness, Vertex-wise cortical thickness, Stroke, Ischemic stroke, Depressive symptoms x independent stressful life events interaction (2df test), Low myopia, Chronic kidney disease (end stage renal disease vs. normal eGFR) in type 1 diabetes, Brain region volumes, Abdominal fat cell number, Migraine, Ceramide (d42:2)B levels, Response to paclitaxel in ovarian cancer (Caspase 3/7 EC50), Ischemic stroke (small-vessel)
cg08637123	chr7	138,764,793	ZC3HAV1	OpenSea	1.78e + 00	3.92e-01	5.92e-06	7.30e-01	systemic sclerosis, age, Tissue	Eosinophil counts, Lymphocyte counts, Platelet count, Body mass index, Immature fraction of reticulocytes, High light scatter reticulocyte percentage of red cells, Reticulocyte fraction of red cells, White blood cell count, Primary biliary cholangitis, White blood cell count, Multiple sclerosis, Eosinophil percentage of white cells, Plateletcrit, Body size at age 10, High light scatter reticulocyte count, Adult body size
cg05819912	chr4	170,945,459	MFAP3L	N_Shore	2.02e + 00	4.47e-01	6.59e-06	7.30e-01	plasma fasting HOMA-IR levels, Tissue, age, HOMA-IR, sex	Educational attainment (years of education), Noncognitive aspects of educational attainment, Educational attainment (MTAG), Calcium levels, Anxiety disorder, Metabolite levels, Highest math class taken (MTAG), Educational attainment, Free thyroxine concentration
cg10737663	chr15	30,865,191	FAM7A2	Island	-9.05e-01	2.03e-01	8.53e-06	7.30e-01	Tissue, age, age, HIV infection	
cg21267754	chr3	11,049,471	SLC6A1	OpenSea	-5.42e + 00	1.22e + 00	9.61e-06	7.30e-01		Refractive error, Lung function, Metabolite levels, Epithelial ovarian cancer, Alcohol consumption x playing computer games interaction, Decanoylcarnitine levels (Biocrates platform), Body fat percentage, Longitudinal alcohol consumption, Conduct disorder (maternal expressed emotions interaction), Response to Vitamin E supplementation, Loneliness (linear analysis)
c. CpG sites associated with ΔLTL at a suggestive level (1e-05)										
CpG	chr	pos	Nearest Gene	Relation to Island	beta	Standard error	*P*-value	fdr	Other EWAS trait association	Other GWAS trait association
cg06152223	chr9	124,464,871	DAB2IP	S_Shelf	6.25e-03	1.20e-03	2.05e-07	1.28e-01		Coronary artery disease, vWF levels, Myocardial infarction, Waist-to-hip ratio adjusted for BMI, Waist-hip index, Nicotine dependence symptom count, Abdominal aortic aneurysm, Prostate cancer (SNP x SNP interaction), Left atrial antero-posterior diameter, Arterial stiffness index, Appendicular lean mass, Heel bone mineral density, Height, Age related hearing loss-related regional glucose metabolism (Bilateral Heschl’s gyrus), Vertex-wise sulcal depth, Coronary artery disease or factor VIII levels, Coronary artery disease, Factor VIII levels or von Willebrand factor levels (pleiotropy), Hip index, Heart rate, Adverse response to chemotherapy (neutropenia/leucopenia) (docetaxel), Type A behavior, Neurofibrillary tangles (SNP x SNP interaction), Thiazide-induced adverse metabolic effects in hypertensive patients, Periodontal microbiota, Metabolite levels (X-11787), Methotrexate-related central neurotoxicity in children treated for acute lymphoblastic leukemia
cg07546684	chr6	149,867,573	PPIL4	Island	1.44e-02	2.85e-03	5.02e-07	1.34e-01	air pollution (PM2.5), Tissue	Rheumatoid arthritis
cg20229027	chr2	95,944,221	PROM2	OpenSea	-9.99e-03	1.98e-03	5.11e-07	1.35e-01	age, Tissue	
cg10574240	chr10	1,158,031	WDR37	OpenSea	-5.18e-03	1.06e-03	1.01e-06	2.17e-01		Total PHF-tau (SNP x SNP interaction), Chronic kidney disease, Cortex volume change rate, interferon-related traits, Glomerular filtration rate in non diabetics (creatinine)
cg07085256	chr21	47,719,456	RPL23AP4	S_Shore	1.01e-02	2.07e-03	1.05e-06	2.26e-01		Cognitive empathy
cg16381191	chr13	113,439,218	ATP11A	N_Shore	-4.68e-03	9.54e-04	1.06e-06	2.30e-01		Protein quantitative trait loci (liver), Red cell distribution width, Mean corpuscular volume, Mean spheric corpuscular volume, High light scatter reticulocyte percentage of red cells, Reticulocyte fraction of red cells, Mean corpuscular hemoglobin, Red blood cell count, Glycated hemoglobin levels, Reticulocyte fraction of red cells, High light scatter reticulocyte count, Reticulocyte count, Cutaneous malignant melanoma, Cutaneous melanoma (MTAG), Red blood cell count, COVID-19 (critical illness vs population or mild symptoms), Machado-Joseph disease (age at onset), Nevus count or cutaneous melanoma, Low tan response, Platelet count, Idiopathic pulmonary fibrosis, TestASV_16 (Bacteroides) prevalence, Basal cell carcinoma, Keratinocyte cancer (MTAG), Interstitial lung disease, Sunburns, Hemoglobin A1c levels
cg07039362	chr16	55,908,870	CES7	OpenSea	-1.16e-02	2.45e-03	2.71e-06	3.98e-01	colon adenocarcinoma survival, age, Tissue, Clear cell renal carcinoma, POMGNT2 protein levels, VAMP7 protein levels	
cg07230786	chr12	113,335,032	RPH3A	OpenSea	-8.97e-03	1.94e-03	3.93e-06	3.98e-01	age, Tissue, Maternal body mass index, Nitrogen dioxide exposure, Age, HIV infection	Urate levels, Systolic blood pressure x smoking status (ever vs never) interaction (2df test), Systolic blood pressure x smoking status (current vs non-current) interaction (2df test), Diastolic blood pressure x smoking status (ever vs never) interaction (2df test), Diastolic blood pressure x smoking status (current vs non-current) interaction (2df test), Low density lipoprotein cholesterol levels, LDL cholesterol levels, Eosinophil counts, Cystatin C levels, Total bilirubin levels, Direct bilirubin levels, Platelet count, Serum uric acid levels, Glycated hemoglobin levels, Serum copper levels, Weight, Alzheimer’s disease polygenic risk score (upper quantile vs lower quantile), Systolic blood pressure, Type 2 diabetes, Diastolic blood pressure, High light scatter reticulocyte count, High light scatter reticulocyte percentage of red cells, Insomnia, Pulse pressure, Body mass index, Apolipoprotein B levels, Tonsillectomy, Lipid traits (pleiotropy) (HIPO component 1), Reaction time, Low HDL-cholesterol levels, Coronary artery disease, Excessive alcohol consumption, Lymphocyte counts, Right unilateral cleft lip and palate, Cholesterol levels in IDL, Cholesteryl ester levels in IDL, Coronary artery disease or factor VII levels (pleiotropy), Omega-6 fatty acid levels, Coronary artery disease or von Willebrand factor levels (pleiotropy), Coronary artery disease or factor XI levels (pleiotropy), Coronary artery disease or tissue plasminogen activator levels (pleiotropy), Height, Total phospholipid levels in lipoprotein particles, Sphingomyelin levels, Total free cholesterol levels, Total cholesterol levels, Total esterified cholesterol levels, Mean spheric corpuscular volume, Alanine aminotransferase levels, Serum alkaline phosphatase levels, Neutrophil-to-lymphocyte ratio, Chloride levels, Mean arterial pressure, Methotrexate pharmacokinetics (acute lymphoblastic leukemia), beta-nerve growth factor levels, Thrombomodulin levels in ischemic stroke, Aerodigestive squamous cell cancer (pleiotropy), Epilepsy, Waist circumference, Spleen volume, Coronary artery disease or fibrinogen levels (pleiotropy), Coronary artery disease or plasminogen activator inhibitor 1 levels (pleiotropy), Smoking status, Mean reticulocyte volume, Lactate levels
cg14514120	chr12	64,801,624	XPOT	S_Shelf	1.01e-02	2.19e-03	4.04e-06	3.98e-01	age, Fetal vs adult liver, Tissue, Gestational age, Rheumatoid arthritis, Alcohol consumption per day, Alcohol consumption, age, MIA protein levels, TFRC protein levels, Alcohol consumption	Mean corpuscular hemoglobin, Endometriosis or asthma (pleiotropy)
cg19060970	chr19	58,038,588	ZNF549	Island	2.48e-02	5.38e-03	4.42e-06	3.98e-01	B Acute Lymphoblastic Leukemia, hepatocellular carcinoma (HCC);aging;vitamin B12 supplement, age, Primary Sjogrens syndrome, Tissue	Male-pattern baldness
cg00325548	chr3	52,947,163	SFMBT1	OpenSea	1.16e-02	2.54e-03	5.08e-06	3.98e-01	eosinophilia, atopy	Waist-to-hip ratio adjusted for BMI, Waist-hip index, Urate levels, Electrocardiogram morphology (amplitude at temporal datapoints), Intelligence (MTAG), Intelligence, General cognitive ability, Educational attainment (years of education), Educational attainment (MTAG), Serum uric acid levels, Height, Estimated glomerular filtration rate, Estimated glomerular filtration rate (creatinine), A body shape index, Waist circumference adjusted for body mass index, Ulcerative colitis, Gout, Positive affect, Life satisfaction, Hip circumference, Mental health study participation (completed survey), Hyperuricemia, Chronic kidney disease, Creatinine levels, Serum levels of protein ITIH3, Cognitive performance (MTAG), Educational attainment, Carnitine levels, Red blood cell count, Stimulated adipocyte lipolysis, Cognitive aspects of educational attainment, Colorectal cancer, Feeling worry, Schizophrenia, Hand grip strength, Disrupted circadian rhythm (low relative amplitude of rest-activity cycles), Haemorrhoidal disease, Weight
cg05622719	chr17	1,532,159	SLC43A2	Island	-2.48e-02	5.42e-03	5.09e-06	3.98e-01		Waist circumference adjusted for body mass index, Appendicular lean mass, Serum albumin levels, Estimated glomerular filtration rate (creatinine), Calcium levels, Reticulocyte fraction of red cells, Mean platelet volume, Plateletcrit, Reticulocyte count
cg24671524	chr14	106,610,373	IGHV7-56	OpenSea	1.34e-02	2.94e-03	5.64e-06	3.98e-01		
cg01721962	chr14	105,421,844	AHNAK2	OpenSea	-7.04e-03	1.55e-03	6.00e-06	3.98e-01		Systemic lupus erythematosus, Circulating levels of total-tau, Serum levels of protein RLN1, Stem cell factor levels
cg06089463	chr4	121,988,066	C4orf31	N_Shelf	-9.14e-03	2.01e-03	6.19e-06	3.98e-01	bicuspid aortic valve (BAV), infertility,mortality, waist circumference (WC), Tissue, Rheumatoid arthritis, sex, age, Total cholesterol to total lipids ratio in large LDL, FSTL1 protein levels, NOTCH1 protein levels, NEGR1 protein levels	
cg14008999	chr17	750,766	NXN	OpenSea	2.90e-02	6.43e-03	7.06e-06	3.98e-01	age, Tissue	Colorectal cancer, Heel bone mineral density, Platelet count, Colorectal cancer or advanced adenoma, Blood pressure, Diastolic blood pressure (long-term average), Waist circumference adjusted for body mass index, Response to paliperidone in schizophrenia (PANSS score), Caudate activity during reward, Facial morphology (factor 15, philtrum width), Emphysema annual change measurement in smokers (adjusted lung density), T cell lymphocyte profile difference, Plateletcrit, Platelet distribution width, Colon polyp, Taxane-induced peripheral neuropathy in breast cancer, Economic and political preferences, Blood pressure, Facial attractiveness (female raters), Breast cancer specific mortality in estrogen receptor positive breast cancer, Methotrexate-related central neurotoxicity in children treated for acute lymphoblastic leukemia, Type 2 diabetes
cg04845060	chr4	53,455,270	LNX1	OpenSea	-2.72e-02	6.06e-03	7.40e-06	3.98e-01	Asthma, air pollution (Na), age, Tissue, Fetal vs adult liver	Pulse pressure, Blood pressure, IDP dMRI TBSS ICVF Retrolenticular part of internal capsule L, IDP dMRI TBSS ICVF Retrolenticular part of internal capsule R, IDP dMRI TBSS ICVF Anterior corona radiata L, IDP dMRI ProbtrackX ICVF ptr r, IDP dMRI ProbtrackX ICVF str l, IDP dMRI ProbtrackX ICVF fma, IDP dMRI TBSS ICVF Posterior thalamic radiation R, IDP dMRI TBSS ICVF Sagittal stratum R, IDP dMRI TBSS ICVF Posterior corona radiata L, IDP dMRI ProbtrackX ICVF ifo r, Uterine fibroids, Systolic blood pressure, Heel bone mineral density, Pulse pressure x alcohol consumption interaction (2df test), Diastolic blood pressure, Body mass index, Male-pattern baldness, Balding type 1, Height, Neutrophil count, Carotid Intima-media thickness (mean of the maximum cIMT), Carotid intima media thickness, Response to paliperidone in schizophrenia (PANSS score), Response to paliperidone in schizophrenia (CGI-S score), Cortical surface area, Vertex-wise cortical surface area, Cortical thickness, Vertex-wise cortical thickness, Descending thoracic aortic diameter, Peginterferon alfa-2a treatment response in chronic hepatitis B infection, Core binding factor acute myeloid leukemia, Coronary artery disease, Metabolite levels, Dementia and core Alzheimer's disease neuropathologic changes, IDP dMRI TBSS L2 Anterior corona radiata L, Aerodigestive squamous cell cancer (pleiotropy), Ischemic stroke in diabetes mellitus, Corneal resistance factor (MTAG), Mean arterial pressure, Central corneal thickness (MTAG), Mean corpuscular hemoglobin, Megamonas abundance in stool, Serum albumin levels, Lung function (FEV1/FVC), Weight, Systolic blood pressure x alcohol consumption interaction (2df test), Pulse pressure x alcohol consumption (light vs heavy) interaction (2df test), Lateral ventricle volume, Cognitive performance (attention) (longitudinal), QT interval, DNA methylation (variation), Lobe attachment (rater-scored or self-reported), Foot ulcer and neuropathy in diabetes, Beard thickness, Hair color, Diffuse plaques (SNP x SNP interaction), Cervical artery dissection, Lung function (FVC), Vertex-wise sulcal depth
cg20222869	chr16	31,475,935	ARMC5	Island	5.32e-03	1.19e-03	7.98e-06	3.98e-01	age	Mean corpuscular volume, Metabolite levels, Glycosuria in pregnancy (maternal and offspring genotype effect), Glycosuria in pregnancy (maternal genotype effect)
cg24579246	chr6	114,289,290	HDAC2	N_Shelf	1.18e-02	2.64e-03	8.12e-06	3.98e-01		
cg02043791	chr8	38,322,629	FGFR1	N_Shore	1.86e-02	4.15e-03	8.24e-06	3.98e-01	B Acute Lymphoblastic Leukemia, age, Tissue, gestational age	Electrocardiogram morphology (amplitude at temporal datapoints), Calcium levels, Body mass index, Waist-hip ratio, Schizophrenia (MTAG), Schizophrenia, Height, Adult body size, Cortical thickness, Vertex-wise cortical thickness, Urate levels, Total testosterone levels, Systolic blood pressure, Autism spectrum disorder, Total PHF-tau (SNP x SNP interaction), PR interval, Bipolar disorder (MTAG), Autism spectrum disorder or schizophrenia, Anorexia nervosa, attention-deficit/hyperactivity disorder, autism spectrum disorder, bipolar disorder, major depression, obsessive–compulsive disorder, schizophrenia, or Tourette syndrome (pleiotropy), Red cell distribution width, Serum total protein level, Triglyceride levels, Waist-to-hip ratio adjusted for BMI, Type 2 diabetes, Body size at age 10, Smoking initiation (ever regular vs never regular) (MTAG), Age of smoking initiation (MTAG), Externalizing behaviour (multivariate analysis), Cortical surface area, Age at first sexual intercourse, Vertex-wise sulcal depth, Lung function (FEV1/FVC), Nonsyndromic cleft lip with cleft palate, Response to cognitive-behavioural therapy in anxiety disorder, Birth weight, Circulating plasma alpha-Klotho levels
cg11838237	chr5	1,752,864	MRPL36	OpenSea	-1.67e-02	3.74e-03	8.70e-06	3.98e-01	adrenocortical carcinoma, hepatocellular carcinoma (HCC), age, Clear cell renal carcinoma, Age 4 vs age 0, IL2RB protein levels, NCR1 protein levels	Core binding factor acute myeloid leukemia, Mosquito bite size, Response to TNF inhibitor in rheumatoid arthritis (change in tender 28-joint count), G_Firmicutes abundance, Average oral glucocorticoid dose in mepolizumab-treated eosinophilic granulomatosis with polyangiitis
cg13487992	chr14	23,511,078	PSMB11	OpenSea	-1.70e-02	3.81e-03	8.81e-06	3.98e-01	adenoma, age, Tissue	Red cell distribution width, CD28 + CD45RA + CD8 + T cell %T cell
cg15107887	chr1	180,469,147	ACBD6	N_Shelf	-1.12e-02	2.51e-03	8.89e-06	3.98e-01	Tissue, age,	
cg06487623	chr17	80,021,247	DUS1L	N_Shore	-8.85e-03	1.99e-03	8.93e-06	3.98e-01		Triacylglycerol 56:6 levels
cg12942052	chr1	22,563,123	EPHA8	OpenSea	-1.53e-02	3.44e-03	9.03e-06	3.98e-01		Serum levels of protein C1QC, Blood protein levels, Serum alkaline phosphatase levels, Interleukin-17 levels, Macrophage colony stimulating factor levels, Interferon gamma levels, Interleukin-8 levels, Tumor necrosis factor beta levels, Educational attainment, Neurofibrillary tangles (SNP x SNP interaction)
cg14465747	chr7	32,111,342	PDE1C	S_Shore	-3.08e-02	6.91e-03	9.15e-06	3.98e-01	Tissue, age, Ulcerative colitis, Gestational age, Inflammatory bowel disease, Papuan ancestry, Crohn's disease	Intelligence (MTAG), Extremely high intelligence, General cognitive ability, Cognitive ability, Intelligence, Body mass index, Educational attainment (years of education), Educational attainment (MTAG), Morningness, Morning person, Chronotype, Cognitive performance, Cognitive performance (MTAG), Sensorimotor dexterity, Total PHF-tau (SNP x SNP interaction), Educational attainment, Smoking behaviour (cigarettes smoked per day), Cigarettes smoked per day (MTAG), Smoking cessation, Smoking cessation (MTAG), Self-reported math ability (MTAG), Highest math class taken (MTAG), Gut microbiota (bacterial taxa, hurdle binary method), Triglyceride levels, Triglycerides, Protein quantitative trait loci (liver), Endometriosis (MTAG), Endometriosis, Cognitive aspects of educational attainment, Neurofibrillary tangles (SNP x SNP interaction), Smoking behaviour (cigarette pack-years), Perceived intensity of glucose, Neuroticism, Smoking behavior, Deep white matter hyperintensities, Adult body size, Externalizing behaviour (multivariate analysis), Age at first sexual intercourse, Body fat percentage, Metabolic biomarkers (multivariate analysis), High-sensitivity cardiac troponin I concentration, Gastroesophageal reflux disease, Weight, Lung function (FEV1/FVC), Gynecologic disease (multivariate analysis), Cognitive ability, years of educational attainment or schizophrenia (pleiotropy), Insomnia, Lifetime smoking index, Predicted visceral adipose tissue, Cognitive ability (MTAG), Verbal-numerical reasoning, Presence of antiphospholipid antibodies, Metabolite levels (HVA/MHPG ratio), Smooth-surface caries, Gout, Palmitic acid (16:0) levels, Caffeine consumption from coffee or tea, Adolescent idiopathic scoliosis, Medication use (thyroid preparations), Abdominal fat cell number, Colorectal cancer
cg21873448	chr8	28,420,427	FZD3	OpenSea	-9.71e-03	2.19e-03	9.82e-06	3.98e-01		Maternal nondisjunction of chromosome 21 (mothers vs fathers), Maternal nondisjunction of chromosome 21 (MII error vs fathers), Long-chain dicarboxylacylcarnitines levels (additive genetic model), Long-chain dicarboxylacylcarnitines levels (dominant genetic model), Anxiety severity x hours spent watching television interaction, Vertex-wise cortical thickness, Vertex-wise cortical surface area, Alcohol consumption x hours spent watching television interaction

**Fig. 3:**
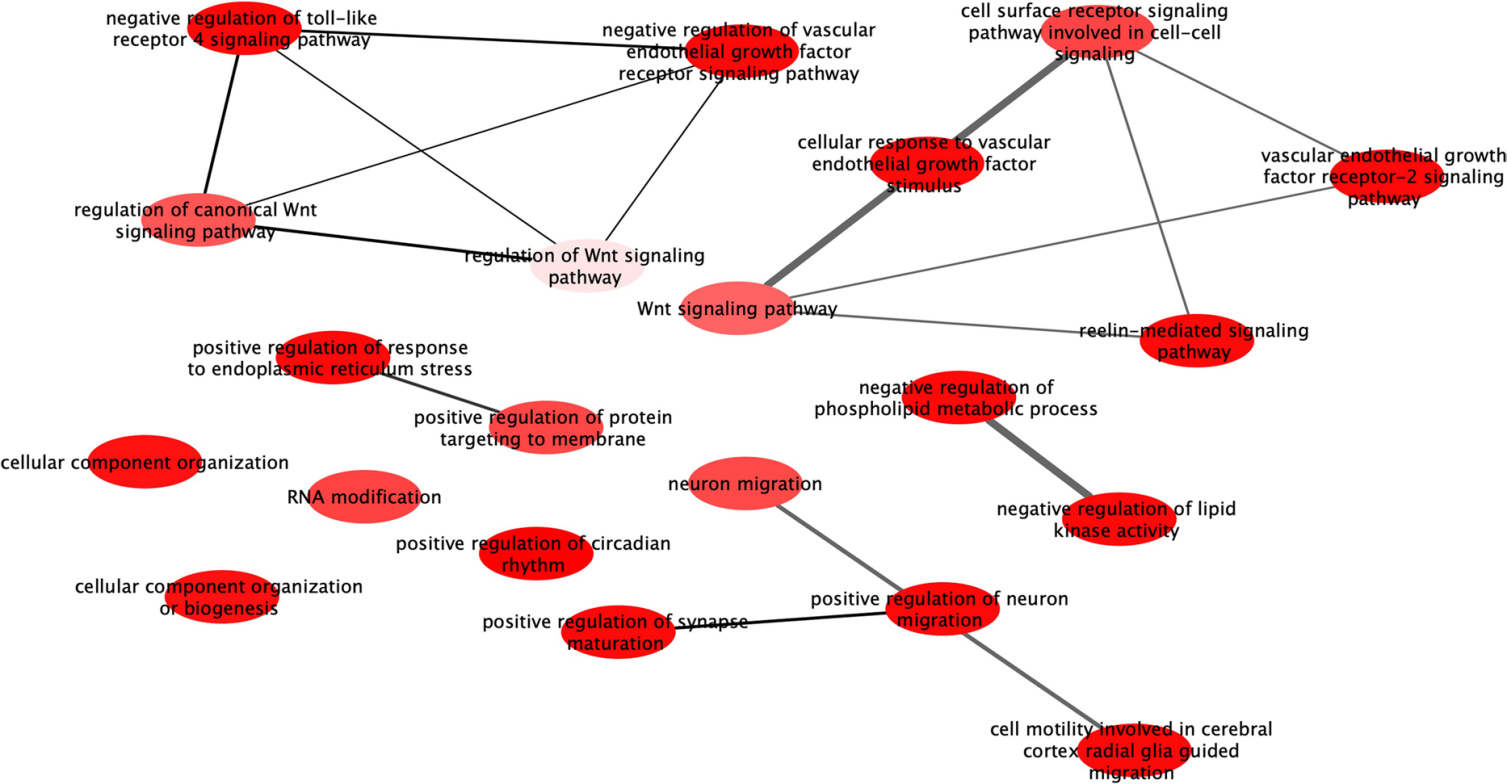
Gene ontology enrichment of nearest genes associated with delta leukocyte telomere length. Red bubble corresponds to *p*-value < 1e-5, pink bubble corresponds to *p*-value < 1e-3. Highly similar GO terms are linked by edges in the graph, where the line width of the edges indicates the degree of similarity. The placement (position and distance) of the nodes indicates the similarity of the GO terms, which is determined by a ‘force-directed’ layout algorithm used in REVIGO tool [66] that aims to keep the more similar nodes closer together

Genes, whose methylation status was associated with** Δ**LTL, were enriched in gene sets involved in the vascular endothelial signaling pathway (i.e. regulation of vascular endothelial growth factor receptor signaling pathway, cell response to vascular endothelial growth factor stimulus and regulation of Wnt signaling pathway), regulation of phospholipid metabolic process and regulation of toll-like receptor 4 signaling pathway (Fig. 3 and Table S2). Gene set was only enriched in the regulation of peptidase activity for measured LTL and regulation of membrane lipid distribution for genetically predicted LTL (Table S2). KEGG pathway analysis revealed that genes linked to **Δ**LTL were related to vascular smooth muscle contraction. However, all the pathways did not survive after multiple comparison corrections (Table S3).

## Discussion

Using a hypothesis-driven approach, we systematically examined the associations of three LTL measures [i.e. measured LTL, genetically predicted LTL, and the difference between the two (**Δ**LTL)] with sensitive quantitative markers of vascular function in the general population. We found that genetically predicted LTL was only associated with the cardiac index. Measured LTL and **Δ**LTL were consistently associated with microvascular function and hemodynamic traits, but not with markers of arterial stiffness or blood pressure. The consistent associations of measured and genetically predicted LTL with cardiac index indicate that longer LTL is likely to be causally related to better cardiac function. Importantly, **Δ**LTL was associated with microvascular function and cardiac index independent of genetically predicted LTL. Of note, **Δ**LTL was more strongly associated with microvascular function and cardiac index than genetically predicted LTL. This suggests that telomere shortening itself, independent of genetic predisposition, contributes to cardiovascular dysfunction [68, 69]. Additionally, genes, whose methylation levels were associated with **Δ**LTL, were enriched in pathways related to vascular endothelial growth factor function and have been linked to environmental exposure, CVDs as well as mortality.

We found that both longer measured LTL as well as higher **Δ**LTL, were associated with better microvascular function. These findings support the notion that telomere shortening could potentially cause microvascular dysfunction, which is an early feature of atherosclerosis and vascular diseases [70]. Although experimental studies have suggested that telomere function is a crucial determinant of microvascular function [44, 49, 71–74], and some studies have shown that telomere length is related to other subclinical markers of atherosclerosis [35, 75, 76], only a few clinical and epidemiological studies have investigated the association of telomere length with microvascular function [28, 29]. One cross-sectional study in 102 patients with a history of cerebrovascular diseases found shortened telomeric 3’-overhang (G-tail), but not telomere length, to be associated with microvascular function [29]. Another cross-sectional study from the LIPGENE cohort including 88 patients with metabolic syndrome also found that microvascular function, through high oxidative stress, was associated with shorter telomere length [28]. Our study, with a larger sample size and a wide age range among community-dwelling adults, not only confirms and substantially extends these previous findings but also provides evidence for a causal connection between shorter telomere length and microvascular dysfunction at the population level.

The relationship of both measured and genetically predicted LTL with hemodynamic indices indicates that telomere shortening may be a biologically important factor that contributes to the age-related decline in heart function. Experimental studies highlighted the important role of cardiac telomere length in heart development, function and disease [77, 78]. Decreases in telomere length in cardiomyocytes induced apoptosis and heart disease [79, 80]. However, there are only a few epidemiological studies assessing the association between LTL and hemodynamic traits. Of note, we found that LTL-related genetic variant mapping to Protection of Telomeres 1 (POT1) was associated with cardiac index. The POT1 protein is one of the six core proteins forming the terminal t-loop shelterin complex of telomere and it is essential for telomere length maintenance and regulation [81]. Previous experimental studies have shown that disruption of POT1 function accelerates telomere shortening, increases apoptosis, and initiates an ATR-dependent DNA damage response [81, 82], which potentially activates immune signaling and chronic inflammation [83]. Moreover, depletion of POT1 has been linked to phagocytosis and nitric oxide generation, which is involved in endothelial dysfunction [84]. Applying a population-based approach in which we leveraged new genetic findings, our findings support a causal role for shorter telomere length in the pathogenesis of cardiac dysfunction across the adult life course.

The molecular processes through which telomere length affects microvascular and cardiac function are thus far poorly understood. Our EWAS and subsequent gene enrichment analyses showed that genes, whose methylation levels were associated with **Δ**LTL, were enriched in pathways related to vascular endothelial growth factor function. Interestingly, the methylation status of these CpGs has been previously linked to environmental exposures, lifestyle factors as well as mortality. Moreover, the mapped genes associated with **Δ**LTL have been related to CVDs and other aging-related phenotypes. These findings have profound implications for our understanding of cardiovascular senescence and suggest that counteracting telomere shortening via non-genetic factors, including nutrition [85, 86], physical activity [69, 87], and sleep [88, 89], may improve microvascular and cardiac function, preventing CVDs independent of the genetic basis of LTL variation.

We found little evidence for an association between LTL measures, arterial stiffness, and blood pressure parameters. Prior studies have observed inconsistent associations between measured or genetically predicted LTL and arterial stiffness and blood pressure traits, with several null results reported [23, 32, 38, 42, 90, 91]. Studies with a larger sample size identified associations between measured/genetically predicted longer LTL with higher blood pressure, but not with arterial stiffness [24, 53]. Moreover, these 197 identified independent genomic variants associated with LTL increased the amount of explained variance in LTL [53]. However, there is an incomplete understanding of the biological underlying mechanism of telomere length with regard to blood pressure traits.

Our study has both strengths and limitations. Strengths of our study include the wide coverage of the vascular phenotypes, the inclusion of individuals across a wide age range from the general population, as well as the availability of estimates of both measured and genetically predicted LTL. This enabled internal validation of the PRS of LTL in our cohort, as well as the derivation of another metric, **Δ**LTL, which allowed estimation of the difference between measured LTL and genetically predicted LTL in relation to vascular phenotypes, independent of genetically predicted LTL. Indeed, we were able to replicate the previously identified top hits related to LTL and were able to construct PRSs of LTL based on SNPs with clear relevance to telomere biology, further supporting the biological plausibility of our findings. The availability of robust PRSs of LTL allowed us to provide evidence for a causal role of telomere length in cardiovascular senescence and dysfunction. Moreover, we performed an EWAS of three LTL measures (i.e. measured LTL, genetically LTL, and **Δ**LTL), which allowed us to further explore the mechanistic insights underlying the role of telomere length in microvascular and cardiac function. A limitation of our study is the lack of longitudinal follow-up data, which precluded the assessment of causality. Moreover, LTL was only measured in a subset of participants, however, there was no significant difference between two datasets. Furthermore, some SNPs previously identified to be associated with LTL were not available on our genetic arrays; however, PRSs created based on two GWAS showed similar results with vascular phenotypes.

In conclusion, both measured and** Δ**TL were consistently associated with microvascular and cardiac function. The association between validated PRSs of LTL and cardiac index supports a causal role for telomere shortening in the pathogenesis of cardiac dysfunction. Importantly, genes, whose methylation levels were associated with **Δ**LTL, were involved in vascular endothelial growth factor function pathways. These findings implicate telomere shortening in the mechanistic pathways underlying cardiovascular dysfunction and CVDs. Combined with prior studies, our data provide evidence that lifestyle interventions may not only reduce the risk of CVDs but also other age-related disorders [92, 93]. These findings also suggest that besides lifestyle interventions that can slow telomere shortening, the development of (pharmacological) treatments that target telomere shortening [94, 95], might improve microvascular and cardiac function independent of the genetic basis of LTL variation, preventing CVDs, and potentially also other age-related diseases.

### Supplementary Information

Below is the link to the electronic supplementary material.

Supplementary file1 (DOCX 1.28 MB)

## Data Availability

The Rhineland Study’s dataset is not publicly available because of data protection regulations. Access to data can be provided to scientists in accordance with the Rhineland Study’s Data Use and Access Policy. Requests for further information or to access the Rhineland Study’s dataset should be directed to RS-DUAC@dzne.de. All the authors had full access to all the data in the study and the corresponding author takes responsibility for data integrity.

## References

[1] Blackburn EH, Epel ES, Lin J (2015). Human telomere biology: A contributory and interactive factor in aging, disease risks, and protection. Science.

[2] Rossiello F (2022). Telomere dysfunction in ageing and age-related diseases. Nat Cell Biol.

[3] Demanelis K, et al. Determinants of telomere length across human tissues. Science. 2020;369(6509):eaaz6876. 10.1126/science.aaz6876 PMC8108546 32913074

[4] Fyhrquist F, Saijonmaa O, Strandberg T (2013). The roles of senescence and telomere shortening in cardiovascular disease. Nat Rev Cardiol.

[5] Li C (2020). Genome-wide association analysis in humans links nucleotide metabolism to leukocyte telomere length. Am J Hum Genet.

[6] Codd V (2022). Measurement and initial characterization of leukocyte telomere length in 474,074 participants in UK Biobank. Nature Aging.

[7] Haycock PC (2014). Leucocyte telomere length and risk of cardiovascular disease: systematic review and meta-analysis. BMJ.

[8] Schneider CV (2022). Association of Telomere Length With Risk of Disease and Mortality. JAMA Intern Med.

[9] Kodali HP, Borrell LN (2021). Telomere length and mortality risk among adults in the United States: The role of age and race and ethnicity. Ann Epidemiol.

[10] Arbeev KG (2020). Association of Leukocyte Telomere Length With Mortality Among Adult Participants in 3 Longitudinal Studies. JAMA Netw Open.

[11] Haycock PC (2017). Association between telomere length and risk of cancer and non-neoplastic diseases: a mendelian randomization study. JAMA Oncol.

[12] Mons U (2017). Leukocyte telomere length and all-cause, cardiovascular disease, and cancer mortality: results from individual-participant-data meta-analysis of 2 large prospective cohort studies. Am J Epidemiol.

[13] Xia K (2021). Leukocyte telomere length and amyotrophic lateral sclerosis: a Mendelian randomization study. Orphanet J Rare Dis.

[14] Gao K (2019). Exploring the causal pathway from telomere length to alzheimer's disease: an update mendelian randomization study. Front Psychiatry.

[15] Scheller Madrid A (2020). Observational and genetic studies of short telomeres and Alzheimer's disease in 67,000 and 152,000 individuals: a mendelian randomization study. Eur J Epidemiol.

[16] Fani L (2020). Telomere length and the risk of alzheimer's disease: the rotterdam study. J Alzheimers Dis.

[17] Fazzini F (2020). Results from the German Chronic Kidney Disease (GCKD) study support association of relative telomere length with mortality in a large cohort of patients with moderate chronic kidney disease. Kidney Int.

[18] Park S (2021). A Mendelian randomization study found causal linkage between telomere attrition and chronic kidney disease. Kidney Int.

[19] D'Mello MJ (2015). Association between shortened leukocyte telomere length and cardiometabolic outcomes: systematic review and meta-analysis. Circ Cardiovasc Genet.

[20] Cheng F (2021). Diabetes, metabolic disease, and telomere length. Lancet Diabetes Endocrinol.

[21] Cheng F (2020). Shortened relative leukocyte telomere length is associated with prevalent and incident cardiovascular complications in type 2 diabetes: analysis from the hong kong diabetes register. Diabetes Care.

[22] Koriath M, et al. Relative telomere length and cardiovascular risk factors. Biomolecules. 2019;9(5):192. 10.3390/biom9050192 PMC6572569 31108918

[23] Rehkopf DH (2016). Leukocyte telomere length in relation to 17 biomarkers of cardiovascular disease risk: a cross-sectional study of US adults. PLoS Med.

[24] Demanelis K, Tong L, Pierce BL (2021). Genetically increased telomere length and aging-related traits in the UK Biobank. J Gerontol A Biol Sci Med Sci..

[25] Chen R (2020). Marital status, telomere length and cardiovascular disease risk in a Swedish prospective cohort. Heart.

[26] Zhan Y (2017). Exploring the causal pathway from telomere length to coronary heart disease: a network mendelian randomization study. Circ Res.

[27] Kuo CL (2019). Telomere length and aging-related outcomes in humans: A Mendelian randomization study in 261,000 older participants. Aging Cell.

[28] Gonzalez-Guardia L (2014). Influence of endothelial dysfunction on telomere length in subjects with metabolic syndrome: LIPGENE study. Age (Dordr).

[29] Nezu T (2015). Telomere G-tail length is a promising biomarker related to white matter lesions and endothelial dysfunction in patients with cardiovascular risk: a cross-sectional study. EBioMedicine.

[30] Vasan RS (2009). Association of leukocyte telomere length with echocardiographic left ventricular mass: the Framingham heart study. Circulation.

[31] Kuznetsova T (2010). Association between left ventricular mass and telomere length in a population study. Am J Epidemiol.

[32] Denil SL (2014). On cross-sectional associations of leukocyte telomere length with cardiac systolic, diastolic and vascular function: the Asklepios study. PLoS One.

[33] Masi S (2014). Rate of telomere shortening and cardiovascular damage: a longitudinal study in the 1946 British Birth Cohort. Eur Heart J.

[34] Honkonen M (2020). Leukocyte telomere length is inversely associated with arterial wave reflection in 566 normotensive and never-treated hypertensive subjects. Aging (Albany NY).

[35] Kosmopoulos M (2022). The relationship between telomere length and putative markers of vascular ageing: A systematic review and meta-analysis. Mech Ageing Dev.

[36] Collerton J (2007). Telomere length is associated with left ventricular function in the oldest old: the Newcastle 85+ study. Eur Heart J.

[37] Peng, H., et al. Impact of biological aging on arterial aging in American Indians: findings from the strong heart family study. Aging (Albany NY). 2016;8(8):1583–92. 10.18632/aging.101013 PMC5032684 27540694

[38] Brown LL (2018). Does telomere length indicate biological, physical, and cognitive health among older adults? evidence from the health and retirement study. J Gerontol A Biol Sci Med Sci.

[39] Loh NY, Noordam R, Christodoulides C (2021). Telomere length and metabolic syndrome traits: a mendelian randomisation study. Aging Cell.

[40] Shen G (2020). The relationship between telomere length and cancer mortality: data from the 1999–2002 national healthy and nutrition examination survey (NHANES). J Nutr Health Aging.

[41] Peng H (2017). Leukocyte telomere length and ideal cardiovascular health in American Indians: the strong heart family study. Eur J Epidemiol.

[42] Bekaert S (2007). Telomere length and cardiovascular risk factors in a middle-aged population free of overt cardiovascular disease. Aging Cell.

[43] Liu Y, Bloom SI, Donato AJ (2019). The role of senescence, telomere dysfunction and shelterin in vascular aging. Microcirculation.

[44] Chen MS, Lee RT, Garbern JC. Senescence mechanisms and targets in the heart. Cardiovasc Res. 2022;118(5):1173–1187. 10.1093/cvr/cvab161 PMC8953446 33963378

[45] Wang J (2015). Vascular smooth muscle cell senescence promotes atherosclerosis and features of plaque vulnerability. Circulation.

[46] Bhayadia R (2016). Senescence-induced oxidative stress causes endothelial dysfunction. J Gerontol A Biol Sci Med Sci.

[47] Hohensinner PJ (2016). Age intrinsic loss of telomere protection via TRF1 reduction in endothelial cells. Biochim Biophys Acta.

[48] Beyer AM (2016). Critical role for telomerase in the mechanism of flow-mediated dilation in the human microcirculation. Circ Res.

[49] Yepuri G (2016). Proton pump inhibitors accelerate endothelial senescence. Circ Res.

[50] Mojiri A (2021). Telomerase therapy reverses vascular senescence and extends lifespan in progeria mice. Eur Heart J.

[51] Njajou OT (2007). Telomere length is paternally inherited and is associated with parental lifespan. Proc Natl Acad Sci U S A.

[52] Broer L (2013). Meta-analysis of telomere length in 19,713 subjects reveals high heritability, stronger maternal inheritance and a paternal age effect. Eur J Hum Genet.

[53] Codd V (2021). Polygenic basis and biomedical consequences of telomere length variation. Nat Genet.

[54] Cawthon RM (2009). Telomere length measurement by a novel monochrome multiplex quantitative PCR method. Nucleic Acids Res.

[55] Salas LA (2022). Enhanced cell deconvolution of peripheral blood using DNA methylation for high-resolution immune profiling. Nat Commun.

[56] Marees AT (2018). A tutorial on conducting genome-wide association studies: quality control and statistical analysis. Int J Methods Psychiatr Res.

[57] Price AL (2006). Principal components analysis corrects for stratification in genome-wide association studies. Nat Genet.

[58] Verma SS (2014). Imputation and quality control steps for combining multiple genome-wide datasets. Front Genet.

[59] Watanabe K (2019). A global overview of pleiotropy and genetic architecture in complex traits. Nat Genet.

[60] Choi PJ (2014). New approach to measure cutaneous microvascular function: an improved test of NO-mediated vasodilation by thermal hyperemia. J Appl Physiol (1985).

[61] Aboyans V (2012). Measurement and interpretation of the ankle-brachial index: a scientific statement from the american heart association. Circulation.

[62] Battram T (2022). The EWAS Catalog: a database of epigenome-wide association studies. Wellcome Open Res.

[63] Li M (2019). EWAS Atlas: a curated knowledgebase of epigenome-wide association studies. Nucleic Acids Res.

[64] Buniello A (2019). The NHGRI-EBI GWAS Catalog of published genome-wide association studies, targeted arrays and summary statistics 2019. Nucleic Acids Res.

[65] Eden E (2009). GOrilla: a tool for discovery and visualization of enriched GO terms in ranked gene lists. BMC Bioinformatics.

[66] Supek F (2011). REVIGO summarizes and visualizes long lists of gene ontology terms. PLoS One.

[67] Phipson B, Maksimovic J, Oshlack A (2016). missMethyl: an R package for analyzing data from Illumina's HumanMethylation450 platform. Bioinformatics.

[68] Yeh JK, Lin MH, Wang CY (2019). Telomeres as therapeutic targets in heart disease. JACC Basic Transl Sci.

[69] Werner CM (2019). Differential effects of endurance, interval, and resistance training on telomerase activity and telomere length in a randomized, controlled study. Eur Heart J.

[70] Davignon J, Ganz P (2004). Role of endothelial dysfunction in atherosclerosis. Circulation..

[71] Minamino T, Mitsialis SA, Kourembanas S (2001). Hypoxia extends the life span of vascular smooth muscle cells through telomerase activation. Mol Cell Biol.

[72] Minamino T (2002). Endothelial cell senescence in human atherosclerosis: role of telomere in endothelial dysfunction. Circulation.

[73] Maeda M, Tsuboi T, Hayashi T (2019). An inhibitor of activated blood coagulation factor x shows anti-endothelial senescence and anti-atherosclerotic effects. J Vasc Res.

[74] Chang E, Harley CB (1995). Telomere length and replicative aging in human vascular tissues. Proc Natl Acad Sci U S A.

[75] Castro-Diehl C (2021). Biomarkers representing key aging-related biological pathways are associated with subclinical atherosclerosis and all-cause mortality: the framingham study. PLoS One.

[76] Fernandez-Alvira JM (2016). Short telomere load, telomere length, and subclinical atherosclerosis: the PESA study. J Am Coll Cardiol.

[77] Booth SA, Charchar FJ (2017). Cardiac telomere length in heart development, function, and disease. Physiol Genomics.

[78] Sharifi-Sanjani M, et al. Cardiomyocyte-specific telomere shortening is a distinct signature of heart failure in humans. J Am Heart Assoc. 2017;6(9):e005086. 10.1161/JAHA.116.005086 PMC5634248 28882819

[79] Anderson R, et al. Length-independent telomere damage drives post-mitotic cardiomyocyte senescence. EMBO J. 2019;38(5):e100492. 10.15252/embj.2018100492 PMC6396144 30737259

[80] Leri A (2003). Ablation of telomerase and telomere loss leads to cardiac dilatation and heart failure associated with p53 upregulation. EMBO J.

[81] Wu L (2006). Pot1 deficiency initiates DNA damage checkpoint activation and aberrant homologous recombination at telomeres. Cell.

[82] He H (2009). Pot1b deletion and telomerase haploinsufficiency in mice initiate an ATR-dependent DNA damage response and elicit phenotypes resembling dyskeratosis congenita. Mol Cell Biol.

[83] Nakad R, Schumacher B (2016). DNA damage response and immune defense: links and mechanisms. Front Genet.

[84] Hagiwara M (2013). POT1b regulates phagocytosis and NO production by modulating activity of the small GTPase Rab5. Biochem Biophys Res Commun.

[85] Leung CW (2018). Diet quality indices and leukocyte telomere length among healthy US adults: data from the national health and nutrition examination survey, 1999–2002. Am J Epidemiol.

[86] Crous-Bou M, Molinuevo JL, Sala-Vila A (2019). Plant-rich dietary patterns, plant foods and nutrients, and telomere length. Adv Nutr.

[87] Tucker LA (2017). Physical activity and telomere length in U.S. men and women: An NHANES investigation. Prev Med.

[88] Tempaku P (2018). Long sleep duration, insomnia, and insomnia with short objective sleep duration are independently associated with short telomere length. J Clin Sleep Med.

[89] Jackowska M (2012). Short sleep duration is associated with shorter telomere length in healthy men: findings from the Whitehall II cohort study. PLoS One.

[90] Nguyen MT (2019). Telomere length and vascular phenotypes in a population-based cohort of children and midlife adults. J Am Heart Assoc.

[91] Eguchi K (2017). Short telomere length is associated with renal impairment in Japanese subjects with cardiovascular risk. PLoS One.

[92] Elks CE, Scott RA (2014). The long and short of telomere length and diabetes. Diabetes.

[93] Sindi S (2021). Telomere length change in a multidomain lifestyle intervention to prevent cognitive decline: a randomized clinical trial. J Gerontol A Biol Sci Med Sci.

[94] Chatterjee S (2021). Telomerase therapy attenuates cardiotoxic effects of doxorubicin. Mol Ther.

[95] Bar C (2014). Telomerase expression confers cardioprotection in the adult mouse heart after acute myocardial infarction. Nat Commun.

