# Supplementary material for: Associations of measured and genetically predicted leukocyte telomere length with vascular phenotypes: a population-based study
Source: GeroScience. 2023 Oct 2;46(2):1947–70. doi: 10.1007/s11357-023-00914-2 (PMC10828293; doi:10.1007/s11357-023-00914-2)
Supplement: Supplementary file 1 — Supplementary file1 (DOCX 1.28 MB) [file 11357_2023_914_MOESM1_ESM.docx]

**Supplementary Materials**

**Associations of Measured and Genetically Predicted Leukocyte Telomere Length with Vascular Phenotypes: A Population-based Study**

**Authors**

Dan Liu, PhD^1^, N. Ahmad Aziz, MD PhD^1,2^, Mohammed Aslam Imtiaz, MSc^1^, Gökhan Pehlivan, MD^1^, Monique M.B. Breteler, MD PhD^1,3*^

**Affiliations**

^1^Population Health Sciences, German Centre for Neurodegenerative Diseases (DZNE), Bonn, Germany

^2^Department of Neurology, Faculty of Medicine, University of Bonn, Bonn, Germany

^3^Institute for Medical Biometry, Informatics and Epidemiology (IMBIE), Faculty of Medicine, University of Bonn, Bonn, Germany

**Figure S1. The correlation between the measured leukocyte telomere length and chronological age**


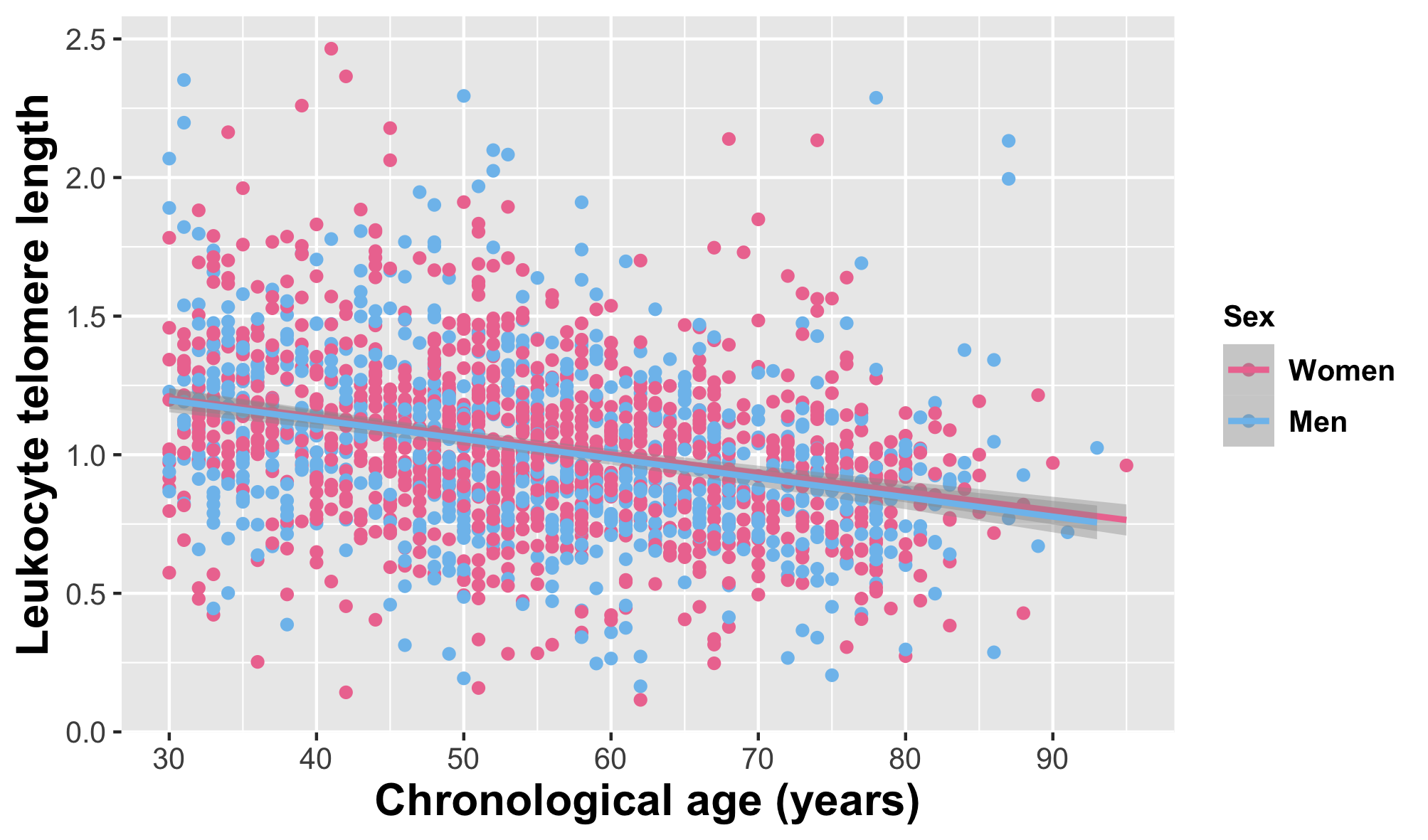


**Figure S2. Correlation matrix between measured leukocyte telomere length (LTL) and the proportion of different leukocyte subtypes**

**
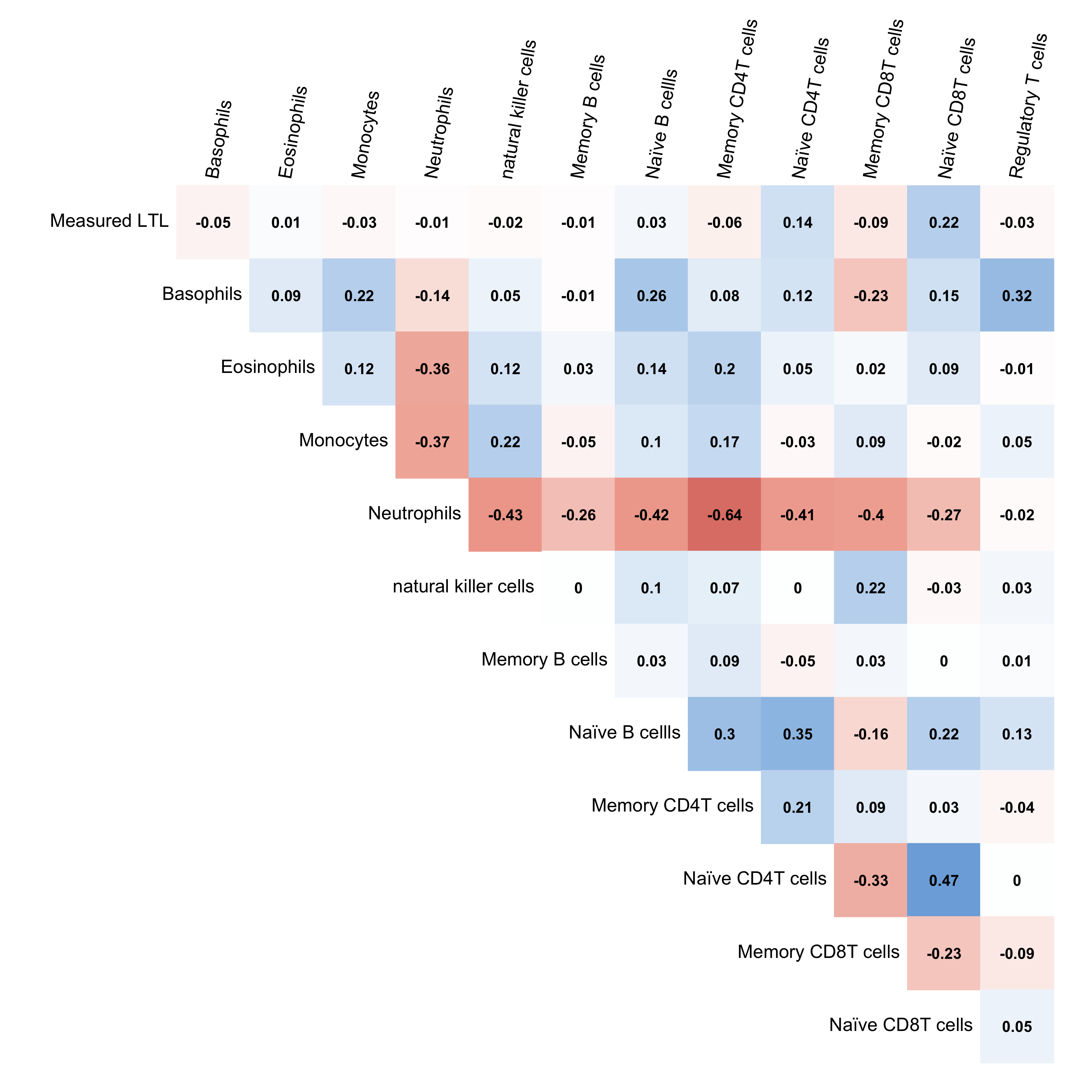
**

**Figure S3. The association between variants associated with leukocyte telomere length and cardiac index**


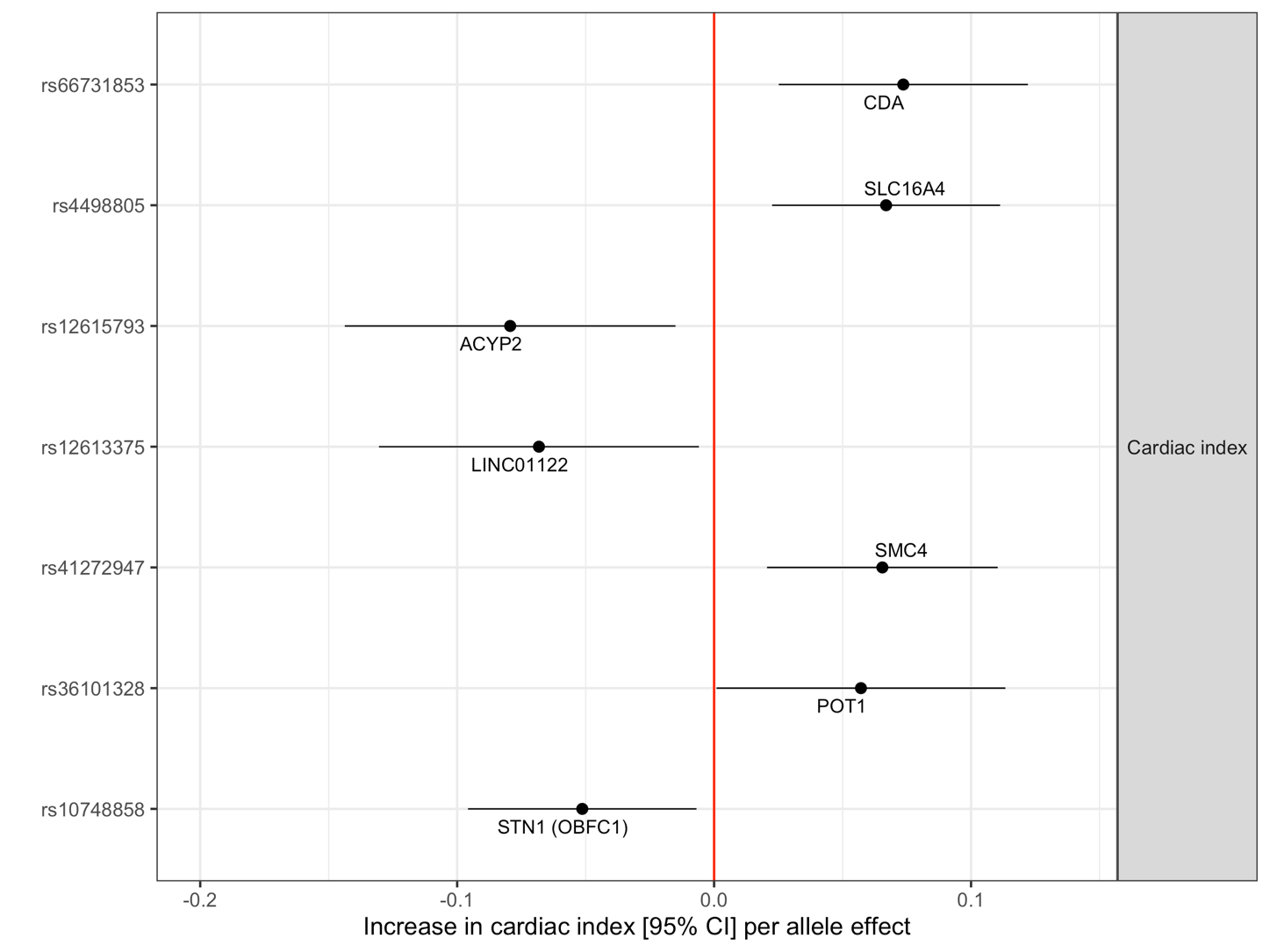


Abbreviation: CI, confidence interval. Analyses were adjusted for age, sex, first ten genetic principal components, smoking status and body mass index.

**Table S1. Genetic variants associated with measured leukocyte** **telomere length in prior genome-wide association studies and replication in the Rhineland Study**

1. **Prior GWAS of measured leukocyte telomere length by Codd et. al 2021**

| **Prior GWAS** | | | | | | **Replication in the Rhineland Study** | | |
| --- | --- | --- | --- | --- | --- | --- | --- | --- |
| **Chr** | **SNP** | **Gene** | **Estimate (95% CI)** | **P value** | **MR#** | **Imputed in RS** | **Estimate (95% CI)** | **P value** |
| ***3*** | ***rs2293607*** | ***TERC*** | ***0.095 (0.090, 0.099)*** | ***1.00e-300*** | ***Yes*** | ***Yes*** | ***0.112 (0.042, 0.181)*** | ***0.002*** |
| 5 | rs7705526 | *TERT* | -0.079 (-0.083, -0.074) | 9.1e-272 | Yes | Yes | -0.039 (-0.113, 0.034) | 0.295 |
| 20 | rs35640778 | *RTEL1* | 0.210 (0.196, 0.223) | 7.00e-190 | Yes | Yes | -0.044 (-0.291, 0.203) | 0.727 |
| **10** | **rs9419958** | ***STN1 (OBFC)*** | **0.081 (0.075, 0.087)** | **6.4e-155** | **Yes** | **Yes** | **0.081 (-0.006, 0.169)** | **0.068** |
| ***5*** | ***rs2853677*** | ***TERT*** | ***0.055 (0.050, 0.059)*** | ***4.8e-150*** | ***No*** | ***Yes*** | ***0.071 (0.008, 0.133)*** | ***0.028*** |
| ***20*** | ***rs2259797*** | ***RTEL1*** | ***0.083 (0.076, 0.090)*** | ***2.5e-115*** | ***No*** | ***Yes*** | ***0.122 (0.01, 0.233)*** | ***0.033*** |
| 4 | rs4435700 | *-F1* | -0.053 (-0.058, -0.048) | 2.5e-102 | Yes | Yes | -0.031 (-0.104, 0.042) | 0.407 |
| ***10*** | ***rs10748858*** | ***STN1 (OBFC1)*** | ***-0.041 (-0.045, -0.037)*** | ***3.3e-84*** | ***No*** | ***Yes*** | ***-0.081 (-0.143, -0.019)*** | ***0.011*** |
| **20** | **rs8114049** | ***RTEL1*** | **0.043 (0.038, 0.047)** | **1.4e-83** | **No** | **Yes** | **0.061 (-0.005, 0.127)** | **0.069** |
| 16 | rs2303262 | *MPHOSPH6* | 0.047 (0.042, 0.052) | 1.8e-81 | Yes | Yes | 0.001 (-0.076, 0.078) | 0.974 |
| 7 | rs7790856 | *POT1* | 0.043 (0.039, 0.048) | 1.8e-80 | Yes | Yes | 0.001 (-0.066, 0.068) | 0.982 |
| 11 | rs611646 | *ATM* | 0.038 (0.033, 0.042) | 1.3e-71 | Yes | No | - | - |
| 18 | rs3891167 | *TYMS* | 0.044 (0.039, 0.049) | 3.7e-71 | Yes | Yes | 0.019 (-0.06, 0.098) | 0.639 |
| 17 | rs4724 | *CTC1* | 0.056 (0.050, 0.062) | 2.1e-64 | Yes | Yes | -0.011 (-0.108, 0.086) | 0.822 |
| 20 | rs1291143 | *SAMHD1* | -0.049 (-0.055, -0.043) | 1.2e-63 | Yes | Yes | -0.041 (-0.127, 0.045) | 0.352 |
| **5** | **rs138895564** | ***TERT*** | **-0.180 (-0.202, -0.158)** | **4.8e-58** | **No** | **Yes** | **-0.409 (-0.862, 0.044)** | **0.076** |
| 2 | rs202034370 | *ACYP2* | 0.106 (0.093, 0.119) | 2.9e-57 | Yes | No | - | - |
| 4 | rs72631678 | *-F1* | -0.033 (-0.037, -0.029) | 2.2e-55 | No | Yes | -0.008 (-0.07, 0.055) | 0.809 |
| 4 | rs113580095 | *-F1* | 0.368 (0.322, 0.415) | 2.9e-55 | No | Yes | 0.300 (-0.492, 1.091) | 0.458 |
| 2 | rs12615793 | *ACYP2* | -0.045 (-0.051, -0.039) | 1.00e-51 | No | Yes | -0.027 (-0.116, 0.062) | 0.554 |
| 20 | rs115610405 | *RTEL1* | 0.111 (0.097, 0.126) | 4.6e-51 | Yes | Yes | 0.076 (-0.182, 0.334) | 0.563 |
| 19 | rs8105767 | *ZNF208* | -0.033 (-0.038, -0.029) | 1.9e-48 | Yes | Yes | -0.056 (-0.123, 0.011) | 0.103 |
| 5 | rs33987166 | *TERT* | -0.097 (-0.110, -0.083) | 5.7e-47 | No | Yes | -0.168 (-0.384, 0.049) | 0.128 |
| 11 | rs141379009 | *ATM* | 0.092 (0.079, 0.104) | 2.8e-46 | No | Yes | -0.062 (-0.249, 0.124) | 0.511 |
| 5 | rs112290073 | *TERT* | -0.139 (-0.158, -0.120) | 5.1e-45 | No | Yes | 0.063 (-0.279, 0.404) | 0.719 |
| 15 | rs181647350 | *ATP8B4* | 0.034 (0.029, 0.039) | 2.6e-44 | Yes | Yes | -0.014 (-0.085, 0.057) | 0.700 |
| ***20*** | ***rs187577818*** | ***RTEL1*** | ***-0.302 (-0.345, -0.260)*** | ***8.6e-44*** | ***Yes*** | ***Yes*** | ***-1.787 (-3.027, -0.548)*** | ***0.005*** |
| 8 | rs10112752 | *TERF1* | 0.029 (0.025, 0.033) | 9.9e-44 | Yes | Yes | 0.011 (-0.051, 0.072) | 0.737 |
| 7 | rs36101328 | *POT1* | 0.031 (0.026, 0.035) | 3.3e-43 | No | Yes | 0.036 (-0.044, 0.115) | 0.380 |
| 14 | rs137901416 | *DCAF4* | -0.045 (-0.052, -0.039) | 1.5e-40 | Yes | Yes | 0.010 (-0.087, 0.108) | 0.838 |
| 1 | rs932002 | *PARP1* | 0.039 (0.033, 0.045) | 2.4e-40 | Yes | Yes | 0.009 (-0.073, 0.09) | 0.837 |
| 18 | rs2741181 | *TYMS* | 0.049 (0.041, 0.056) | 3.7e-40 | No | Yes | 0.011 (-0.099, 0.121) | 0.844 |
| 2 | rs775145631 | *UNC80* | 0.027 (0.023, 0.031) | 4.2e-38 | Yes | No | - | - |
| 6 | rs55965437 | *PRRC2A* | 0.028 (0.024, 0.032) | 4.2e-38 | No | No | - | - |
| 1 | rs3838300 | *MAGI3* | 0.033 (0.028, 0.038) | 2.9e-35 | Yes | Yes | 0.046 (-0.03, 0.122) | 0.231 |
| 20 | rs41308088 | *RTEL1* | -0.047 (-0.054, -0.039) | 1.6e-34 | No | Yes | 0.010 (-0.093, 0.113) | 0.854 |
| 7 | rs117811540 | *POT1* | -0.145 (-0.168, -0.121) | 1.3e-33 | No | Yes | -0.065 (-0.382, 0.252) | 0.688 |
| 20 | rs187013287 | *RTEL1* | 0.275 (0.230, 0.320) | 2.1e-33 | Yes | Yes | 0.789 (-0.221, 1.8) | 0.126 |
| 16 | rs11866592 | *RFWD3* | -0.034 (-0.040, -0.028) | 3.7e-31 | Yes | Yes | -0.039 (-0.129, 0.052) | 0.399 |
| 18 | rs2276182 | *POLI* | -0.024 (-0.028, -0.020) | 2.6e-30 | Yes | No | - | - |
| 9 | rs11557154 | *DCAF12* | 0.035 (0.029, 0.041) | 3.6e-30 | No | Yes | 0.037 (-0.052, 0.126) | 0.413 |
| 16 | rs62046862 | *BANP* | -0.024 (-0.028, -0.020) | 3.6e-30 | Yes | Yes | -0.052 (-0.115, 0.011) | 0.104 |
| **20** | **rs6030416** | ***SAMHD1*** | **0.033 (0.027, 0.039)** | **2.00e-28** | **No** | **Yes** | **0.073 (-0.012, 0.159)** | **0.090** |
| 10 | rs182641927 | *STN1 (OBFC)* | -0.195 (-0.229, -0.160) | 3.3e-28 | No | Yes | -0.037 (-0.754, 0.679) | 0.919 |
| 10 | 10:101274251_CT_C | *NKX2-3* | 0.023 (0.019, 0.028) | 3.7e-28 | Yes | No | - | - |
| 14 | rs1007934 | *DCAF4* | 0.023 (0.019, 0.027) | 3.7e-28 | No | Yes | 0.017 (-0.046, 0.079) | 0.603 |
| ***18*** | ***rs111811424*** | ***TYMS*** | ***-0.040 (-0.047, -0.033)*** | ***5.2e-28*** | ***No*** | ***Yes*** | ***-0.141 (-0.249, -0.034)*** | ***0.010*** |
| 5 | rs34094720 | *TERT* | -0.155 (-0.183, -0.127) | 8.00e-28 | No | Yes | 0.346 (-0.181, 0.874) | 0.198 |
| 17 | rs144204502 | *TK1* | 0.101 (0.083, 0.120) | 3.4e-27 | Yes | Yes | 0.036 (-0.219, 0.291) | 0.782 |
| 10 | rs12572897 | *NOC3L* | 0.034 (0.028, 0.040) | 4.00e-27 | Yes | Yes | 0.030 (-0.064, 0.123) | 0.536 |
| 4 | rs9990898 | *-F1* | -0.032 (-0.038, -0.026) | 6.6e-27 | No | Yes | -0.051 (-0.14, 0.038) | 0.257 |
| 8 | rs762679 | *MCM4* | -0.031 (-0.037, -0.025) | 4.3e-26 | Yes | Yes | 0.026 (-0.058, 0.111) | 0.537 |
| **18** | **rs79824385** | ***LINC01478*** | **-0.032 (-0.038, -0.026)** | **4.3e-26** | **No** | **Yes** | **-0.080 (-0.17, 0.010)** | **0.080** |
| ***11*** | ***rs939916*** | ***ODF3*** | ***-0.024 (-0.028, -0.019)*** | ***6.3e-26*** | ***Yes*** | ***Yes*** | ***-0.081 (-0.15, -0.012)*** | ***0.022*** |
| 16 | rs7193541 | *RFWD3* | 0.022 (0.018, 0.026) | 7.2e-26 | No | Yes | 0.018 (-0.043, 0.079) | 0.564 |
| 18 | rs9952504 | *TYMS* | 0.058 (0.047, 0.069) | 2.8e-25 | No | Yes | 0.092 (-0.049, 0.234) | 0.200 |
| 22 | rs131796 | *TYMP* | 0.025 (0.020, 0.030) | 5.2e-25 | Yes | No | - | - |
| 18 | rs8088824 | *LINC01478* | -0.025 (-0.030, -0.020) | 5.6e-25 | Yes | Yes | -0.043 (-0.115, 0.028) | 0.235 |
| 16 | rs528301822 | *TERF2* | -0.023 (-0.028, -0.019) | 2.4e-24 | Yes | No | - | - |
| 22 | rs28502153 | *GAB4* | 0.022 (0.017, 0.026) | 3.6e-24 | Yes | Yes | 0.031 (-0.034, 0.096) | 0.344 |
| 8 | rs2306646 | *XPO7* | 0.021 (0.017, 0.025) | 4.2e-24 | Yes | Yes | 0.012 (-0.049, 0.073) | 0.697 |
| 15 | rs17677991 | *MGA* | -0.022 (-0.026, -0.018) | 7.8e-24 | Yes | Yes | 0.007 (-0.055, 0.07) | 0.820 |
| 23 | rs782080341 | *MAGEA6* | -0.023 (-0.028, -0.019) | 1.1e-23 | No | No | - | - |
| 7 | rs1985369 | *VIPR2* | 0.031 (0.025, 0.037) | 2.4e-23 | Yes | Yes | 0.031 (-0.062, 0.123) | 0.512 |
| 15 | rs5742915 | *PML* | -0.020 (-0.024, -0.016) | 7.8e-23 | No | Yes | -0.040 (-0.1, 0.021) | 0.201 |
| 5 | rs61748181 | *TERT* | 0.059 (0.047, 0.071) | 1.4e-22 | Yes | Yes | 0.023 (-0.156, 0.201) | 0.805 |
| **16** | **rs62053340** | ***EXOSC6*** | **0.021 (0.017, 0.025)** | **1.5e-22** | **Yes** | **Yes** | **0.061 (-0.004, 0.126)** | **0.064** |
| 3 | rs78491606 | *SHQ1* | 0.073 (0.059, 0.088) | 1.7e-22 | Yes | Yes | 0.068 (-0.176, 0.312) | 0.585 |
| **17** | **rs75664430** | ***CTC1*** | **0.023 (0.018, 0.028)** | **2.3e-22** | **Yes** | **Yes** | **0.061 (-0.01, 0.131)** | **0.091** |
| 3 | rs146546514 | *TERC* | -0.082 (-0.099, -0.065) | 3.8e-22 | No | Yes | 0.126 (-0.188, 0.439) | 0.432 |
| 7 | rs4731541 | *TNPO3* | 0.020 (0.016, 0.025) | 1.6e-21 | Yes | Yes | 0.016 (-0.046, 0.077) | 0.621 |
| 17 | rs7218033 | *RPA1* | 0.023 (0.018, 0.027) | 5.1e-21 | Yes | Yes | -0.028 (-0.099, 0.043) | 0.443 |
| 17 | rs12941945 | *BRCA1* | 0.026 (0.021, 0.032) | 7.1e-21 | Yes | Yes | -0.004 (-0.085, 0.077) | 0.926 |
| 17 | rs5030755 | *RPA1* | -0.030 (-0.036, -0.024) | 8.00e-21 | No | Yes | 0.064 (-0.034, 0.161) | 0.200 |
| 5 | 5:1303867_CT_C | *TERT* | -0.141 (-0.171, -0.111) | 2.1e-20 | No | No | - | - |
| 14 | rs762810 | *MAX* | 0.020 (0.016, 0.024) | 3.9e-20 | Yes | Yes | 0.037 (-0.027, 0.102) | 0.258 |
| 8 | rs564224004 | *TGS1* | 0.030 (0.023, 0.036) | 1.2e-19 | Yes | No | - | - |
| 7 | rs2056726 | *STAG3* | 0.023 (0.018, 0.027) | 1.7e-19 | Yes | Yes | 0.003 (-0.07, 0.077) | 0.928 |
| 10 | rs762222726 | *ASB13* | 0.019 (0.015, 0.023) | 7.3e-19 | Yes | No | - | - |
| 14 | 14:91970514_GA_G | *PPP4R3A* | -0.018 (-0.022, -0.014) | 8.1e-19 | Yes | No | - | - |
| 1 | rs6669563 | *SPOCD1* | -0.018 (-0.023, -0.014) | 8.9e-19 | Yes | Yes | -0.031 (-0.093, 0.03) | 0.319 |
| 12 | rs76666449 | *SRSF9* | -0.030 (-0.037, -0.023) | 1.2e-18 | Yes | Yes | -0.073 (-0.168, 0.021) | 0.129 |
| 4 | rs871134 | *CCDC96* | 0.018 (0.014, 0.022) | 2.2e-18 | Yes | Yes | -0.018 (-0.081, 0.044) | 0.560 |
| 18 | rs116863223 | *TYMS* | 0.082 (0.064, 0.101) | 4.1e-18 | No | Yes | -0.186 (-0.468, 0.096) | 0.195 |
| 16 | rs139438549 | *ACD* | -0.280 (-0.343, -0.216) | 5.7e-18 | Yes | Yes | 0.230 (-0.651, 1.11) | 0.609 |
| 16 | rs182059586 | *PARN* | 0.059 (0.046, 0.073) | 8.6e-18 | Yes | Yes | 0.148 (-0.127, 0.423) | 0.292 |
| **13** | **rs1332941** | ***KBTBD6*** | **-0.024 (-0.030, -0.019)** | **2.1e-17** | **Yes** | **Yes** | **-0.077 (-0.16, 0.006)** | **0.068** |
| 3 | rs6776756 | *GATA2* | 0.018 (0.014, 0.022) | 2.7e-17 | Yes | Yes | -0.004 (-0.067, 0.059) | 0.909 |
| 16 | rs35216338 | *BANP* | -0.044 (-0.054, -0.034) | 2.8e-17 | No | Yes | -0.058 (-0.241, 0.125) | 0.534 |
| 20 | rs577449057 | *RTEL1* | 0.154 (0.119, 0.190) | 2.8e-17 | Yes | No | - | - |
| 5 | rs575928023 | *TERT* | -0.089 (-0.110, -0.069) | 3.4e-17 | No | No | - | - |
| 12 | rs1727302 | *MPHOSPH9* | -0.020 (-0.025, -0.016) | 3.5e-17 | No | Yes | -0.012 (-0.081, 0.058) | 0.742 |
| 5 | rs115451758 | *TERT* | 0.085 (0.066, 0.105) | 3.7e-17 | No | Yes | 0.056 (-0.281, 0.394) | 0.744 |
| 12 | rs79977579 | *SMUG1* | -0.029 (-0.036, -0.022) | 4.8e-17 | Yes | No | - | - |
| 23 | rs147424714 | *mir223* | -0.062 (-0.077, -0.048) | 6.7e-17 | No | No | - | - |
| 10 | rs4919611 | *PPRC1* | 0.027 (0.021, 0.034) | 1.00e-16 | Yes | Yes | 0.024 (-0.067, 0.115) | 0.603 |
| 5 | rs34255404 | *UBE2D2* | -0.038 (-0.047, -0.029) | 1.1e-16 | Yes | No | - | - |
| 6 | rs80324517 | *LOC285766* | -0.040 (-0.049, -0.030) | 1.1e-16 | Yes | Yes | -0.060 (-0.195, 0.076) | 0.388 |
| 7 | rs117247304 | *LOC102723672* | 0.065 (0.049, 0.081) | 3.1e-16 | Yes | No | - | - |
| 1 | rs66731853 | *CDA* | 0.018 (0.014, 0.022) | 3.3e-16 | Yes | Yes | -0.035 (-0.101, 0.03) | 0.289 |
| 3 | 3:49959570_CA_C | *MST1R* | 0.017 (0.013, 0.021) | 3.8e-16 | No | No | - | - |
| 16 | rs76219171 | *PAPD5* | -0.036 (-0.044, -0.027) | 4.7e-16 | Yes | Yes | -0.045 (-0.169, 0.079) | 0.478 |
| 17 | rs62079650 | *BRCA1* | 0.023 (0.017, 0.028) | 1.7e-15 | No | Yes | 0.037 (-0.095, 0.168) | 0.584 |
| 3 | rs41272947 | *SMC4* | 0.016 (0.012, 0.021) | 1.9e-15 | Yes | Yes | 0.045 (-0.017, 0.106) | 0.157 |
| 8 | rs540491189 | *TERF1* | -0.188 (-0.234, -0.141) | 2.7e-15 | Yes | No | - | - |
| 20 | rs112802859 | *SAMHD1* | -0.028 (-0.035, -0.021) | 3.7e-15 | No | Yes | 0.042 (-0.068, 0.152) | 0.451 |
| 16 | rs142507451 | *ACD* | 0.151 (0.113, 0.189) | 8.00e-15 | Yes | Yes | 0.270 (-0.516, 1.056) | 0.500 |
| 13 | rs35017269 | *DIS3* | -0.065 (-0.082, -0.049) | 1.1e-14 | Yes | Yes | 0.032 (-0.204, 0.268) | 0.790 |
| 5 | rs113206288 | *TERT* | -0.023 (-0.028, -0.017) | 1.3e-14 | No | Yes | -0.037 (-0.128, 0.053) | 0.423 |
| 16 | rs80116508 | *SLX4* | 0.033 (0.024, 0.041) | 2.00e-14 | Yes | No | - | - |
| 23 | rs4898396 | *DKC1* | 0.014 (0.011, 0.018) | 3.1e-14 | No | No | - | - |
| **5** | **rs79717857** | ***TERT*** | **-0.051 (-0.064, -0.038)** | **3.2e-14** | **No** | **Yes** | **-0.180 (-0.377, 0.016)** | **0.072** |
| 8 | rs1023767 | *VIRMA* | 0.018 (0.014, 0.023) | 4.3e-14 | Yes | Yes | -0.002 (-0.071, 0.067) | 0.950 |
| 12 | rs10774625 | *SH2B3* | -0.016 (-0.020, -0.012) | 4.3e-14 | No | Yes | -0.001 (-0.062, 0.06) | 0.985 |
| 9 | rs4742448 | *DMRT1* | -0.016 (-0.020, -0.012) | 5.5e-14 | Yes | No | - | - |
| 1 | rs4498805 | *SLC16A4* | -0.015 (-0.019, -0.011) | 1.00e-13 | Yes | Yes | -0.011 (-0.072, 0.05) | 0.729 |
| 3 | rs2811491 | *GATA2* | -0.016 (-0.020, -0.012) | 1.00e-13 | No | Yes | -0.048 (-0.109, 0.013) | 0.122 |
| 5 | rs112951499 | *LOC105374602* | -0.034 (-0.043, -0.025) | 1.3e-13 | Yes | Yes | -0.059 (-0.217, 0.099) | 0.465 |
| 7 | rs138061125 | *VIPR2* | -0.044 (-0.056, -0.032) | 2.00e-13 | No | Yes | 0.004 (-0.207, 0.215) | 0.970 |
| 10 | rs11190184 | *NKX2-3* | 0.017 (0.012, 0.021) | 3.5e-13 | No | Yes | 0.018 (-0.051, 0.087) | 0.615 |
| 6 | rs154979 | *HLA-DMB* | -0.044 (-0.056, -0.032) | 3.9e-13 | No | Yes | 0.417 (0.128, 0.706) | 0.005 |
| 4 | rs13129697 | *SLC2A9* | -0.017 (-0.021, -0.012) | 5.2e-13 | Yes | Yes | -0.014 (-0.082, 0.054) | 0.683 |
| 16 | rs8053839 | *LONP2* | 0.015 (0.011, 0.019) | 5.9e-13 | Yes | Yes | -0.017 (-0.078, 0.043) | 0.579 |
| 12 | rs4758644 | *ZCCHC8* | 0.017 (0.012, 0.022) | 6.9e-13 | Yes | Yes | 0.001 (-0.068, 0.071) | 0.973 |
| 14 | rs73581419 | *RAB2B* | -0.024 (-0.030, -0.017) | 7.6e-13 | Yes | Yes | -0.029 (-0.138, 0.079) | 0.597 |
| 14 | rs1957937 | *TCL1A* | -0.020 (-0.026, -0.015) | 7.8e-13 | Yes | Yes | -0.019 (-0.106, 0.068) | 0.663 |
| 19 | rs8102497 | *PEG3* | 0.015 (0.011, 0.019) | 8.00e-13 | Yes | Yes | 0.024 (-0.037, 0.086) | 0.440 |
| 22 | rs5845706 | *SMC1B* | -0.015 (-0.020, -0.011) | 1.7e-12 | Yes | Yes | 0.042 (-0.022, 0.106) | 0.201 |
| 16 | rs9923119 | *PRDM7* | -0.018 (-0.022, -0.013) | 1.9e-12 | Yes | Yes | 0.019 (-0.053, 0.092) | 0.602 |
| **16** | **rs183553155** | ***RFWD3*** | **-0.072 (-0.093, -0.052)** | **2.00e-12** | **No** | **Yes** | **-0.222 (-0.464, 0.019)** | **0.071** |
| 3 | rs575032615 | *SMARCC1* | -0.062 (-0.079, -0.045) | 2.1e-12 | Yes | No | - | - |
| 7 | rs13230646 | *STK31* | 0.017 (0.012, 0.021) | 2.3e-12 | Yes | Yes | 0.043 (-0.027, 0.113) | 0.227 |
| 2 | rs9752694 | *SMC6* | 0.015 (0.011, 0.019) | 2.4e-12 | Yes | No | - | - |
| 4 | rs4695407 | *OCIAD1* | -0.014 (-0.019, -0.010) | 2.4e-12 | No | Yes | -0.040 (-0.101, 0.021) | 0.200 |
| ***16*** | ***rs11646283*** | ***USP7*** | ***-0.015 (-0.019, -0.011)*** | ***2.4e-12*** | ***Yes*** | ***Yes*** | ***-0.065 (-0.127, -0.002)*** | ***0.042*** |
| 1 | rs61818036 | *PSMB4* | 0.020 (0.014, 0.025) | 3.4e-12 | Yes | Yes | -0.008 (-0.091, 0.074) | 0.842 |
| 2 | rs56178008 | *TRMT61B* | -0.014 (-0.019, -0.010) | 3.8e-12 | Yes | No | - | - |
| 1 | rs139795227 | *RPAP2* | -0.061 (-0.078, -0.044) | 4.9e-12 | Yes | Yes | -0.157 (-0.434, 0.119) | 0.263 |
| 17 | rs1143697 | *TK1* | -0.014 (-0.018, -0.010) | 5.5e-12 | No | Yes | 0.002 (-0.059, 0.063) | 0.953 |
| 3 | 3:138398778_TA_T | *PIK3CB* | -0.014 (-0.019, -0.010) | 6.6e-12 | Yes | No | - | - |
| 20 | 20:62321690_GAGA_G | *RTEL1* | -0.122 (-0.156, -0.087) | 6.7e-12 | Yes | No | - | - |
| 4 | rs753936006 | *POLN* | 0.024 (0.017, 0.030) | 7.3e-12 | Yes | No | - | - |
| 20 | rs544699357 | *SRSF6* | -0.172 (-0.222, -0.123) | 8.1e-12 | Yes | No | - | - |
| 3 | rs869785 | *THRB* | 0.015 (0.011, 0.019) | 9.2e-12 | Yes | Yes | -0.056 (-0.119, 0.007) | 0.080 |
| 1 | rs11579626 | *CHD1L* | -0.025 (-0.032, -0.018) | 1.00e-11 | Yes | Yes | 0.026 (-0.082, 0.134) | 0.639 |
| 6 | rs9398196 | *CCDC162P* | 0.014 (0.010, 0.018) | 1.1e-11 | No | Yes | 0.027 (-0.035, 0.088) | 0.394 |
| 5 | 5:78951569_GT_G | *TENT2 (PAPD4)* | 0.024 (0.017, 0.031) | 1.2e-11 | Yes | No | - | - |
| 11 | rs10840270 | *WEE1* | -0.015 (-0.019, -0.011) | 1.4e-11 | Yes | Yes | -0.029 (-0.093, 0.034) | 0.366 |
| 14 | rs12884911 | *PPP1R36* | 0.014 (0.010, 0.018) | 3.00e-11 | Yes | Yes | -0.009 (-0.07, 0.053) | 0.784 |
| 12 | rs10845387 | *LINC01252* | 0.014 (0.010, 0.019) | 4.6e-11 | Yes | Yes | 0.001 (-0.064, 0.066) | 0.977 |
| 7 | rs11769630 | *IKZF1* | 0.026 (0.018, 0.034) | 4.8e-11 | Yes | Yes | 0.020 (-0.09, 0.131) | 0.718 |
| 4 | rs35500378 | *EXOSC9* | 0.014 (0.010, 0.018) | 1.00e-10 | Yes | No | - | - |
| 20 | rs3761121 | *RTEL1* | -0.020 (-0.026, -0.014) | 1.1e-10 | No | Yes | -0.078 (-0.177, 0.02) | 0.120 |
| 2 | rs12613375 | *LINC01122* | -0.019 (-0.025, -0.013) | 1.4e-10 | Yes | Yes | 0.067 (-0.021, 0.156) | 0.135 |
| 9 | rs4743037 | *ZNF462* | -0.016 (-0.021, -0.011) | 1.4e-10 | Yes | Yes | 0.049 (-0.022, 0.121) | 0.173 |
| 3 | rs201009932 | *SMC4* | 0.076 (0.053, 0.099) | 1.7e-10 | No | Yes | -0.039 (-0.34, 0.261) | 0.797 |
| 1 | 1:41236837_CT_C | *NFYC* | -0.016 (-0.021, -0.011) | 1.8e-10 | Yes | No | - | - |
| 16 | rs34003787 | *ZFHX3* | 0.024 (0.016, 0.031) | 1.8e-10 | Yes | Yes | 0.082 (-0.032, 0.197) | 0.158 |
| 19 | rs35601737 | *TRMT1* | 0.014 (0.010, 0.019) | 1.8e-10 | Yes | Yes | 0.004 (-0.061, 0.07) | 0.897 |
| 17 | rs2069536 | *TEN1* | 0.015 (0.010, 0.020) | 2.2e-10 | Yes | Yes | 0.015 (-0.055, 0.084) | 0.675 |
| 12 | rs79228077 | *MUC8* | 0.015 (0.010, 0.019) | 2.8e-10 | Yes | Yes | 0.041 (-0.042, 0.123) | 0.334 |
| 11 | rs2293579 | *PSMC3* | 0.013 (0.009, 0.018) | 2.9e-10 | No | Yes | 0.022 (-0.045, 0.09) | 0.511 |
| 20 | rs111527478 | *RTEL1* | -0.023 (-0.030, -0.016) | 2.9e-10 | Yes | Yes | -0.024 (-0.134, 0.086) | 0.672 |
| 15 | rs1980240 | *TEX9* | -0.013 (-0.017, -0.009) | 4.7e-10 | Yes | Yes | -0.045 (-0.106, 0.016) | 0.147 |
| 7 | rs609953 | *RNU6-2* | -0.013 (-0.017, -0.009) | 5.5e-10 | Yes | Yes | -0.043 (-0.106, 0.02) | 0.179 |
| 7 | rs11556924 | *ZC3HC1* | -0.013 (-0.017, -0.009) | 5.6e-10 | No | Yes | -0.016 (-0.078, 0.046) | 0.609 |
| 3 | rs13062095 | *SENP7* | -0.014 (-0.018, -0.009) | 5.7e-10 | No | Yes | 0.026 (-0.038, 0.09) | 0.418 |
| 19 | rs80337039 | *MAP2K2* | -0.085 (-0.112, -0.058) | 6.9e-10 | Yes | Yes | -0.350 (-0.934, 0.233) | 0.239 |
| 23 | rs34014897 | *ATRX* | 0.011 (0.008, 0.015) | 8.4e-10 | No | No | - | - |
| 18 | rs1879100 | *PARD6G* | 0.019 (0.013, 0.025) | 9.3e-10 | Yes | Yes | -0.031 (-0.123, 0.061) | 0.512 |
| 19 | rs4530278 | *CEBPA* | -0.013 (-0.017, -0.009) | 9.6e-10 | Yes | Yes | 0.052 (-0.01, 0.114) | 0.099 |
| 14 | rs3093888 | *TEP1* | 0.029 (0.019, 0.038) | 1.1e-09 | Yes | Yes | 0.019 (-0.129, 0.166) | 0.801 |
| 1 | rs17185038 | *RPA2* | 0.025 (0.017, 0.033) | 1.2e-09 | Yes | Yes | -0.034 (-0.191, 0.124) | 0.673 |
| 5 | rs115251750 | *TERT* | -0.034 (-0.045, -0.023) | 1.2e-09 | No | Yes | 0.072 (-0.125, 0.269) | 0.471 |
| 17 | rs111527438 | *ADAP2* | -0.013 (-0.017, -0.009) | 1.7e-09 | Yes | Yes | -0.032 (-0.096, 0.033) | 0.338 |
| 19 | rs429358 | *APOE* | -0.017 (-0.023, -0.011) | 2.3e-09 | No | Yes | -0.010 (-0.099, 0.079) | 0.825 |
| 12 | rs12369950 | *LINC00477* | 0.018 (0.012, 0.024) | 2.5e-09 | Yes | Yes | 0.048 (-0.049, 0.146) | 0.331 |
| 5 | rs72801474 | *HSPA4* | 0.021 (0.014, 0.028) | 2.6e-09 | No | Yes | -0.058 (-0.163, 0.047) | 0.282 |
| 1 | rs41269079 | *BEST4* | -0.015 (-0.021, -0.010) | 3.6e-09 | Yes | Yes | -0.047 (-0.122, 0.028) | 0.217 |
| 16 | rs529549411 | *EXOSC6* | 0.090 (0.060, 0.119) | 4.7e-09 | No | No | - | - |
| 7 | rs2538745 | *UPK3B* | 0.012 (0.008, 0.016) | 4.8e-09 | Yes | No | - | - |
| 1 | rs187540244 | *EXOSC10* | 0.088 (0.059, 0.118) | 4.9e-09 | Yes | Yes | -0.126 (-1.008, 0.757) | 0.780 |
| 1 | rs3768321 | *PABPC4* | 0.015 (0.010, 0.020) | 5.00e-09 | No | Yes | 0.032 (-0.043, 0.108) | 0.399 |
| 16 | rs28711261 | *ACD* | -0.018 (-0.024, -0.012) | 5.9e-09 | No | Yes | -0.057 (-0.147, 0.032) | 0.207 |
| 11 | rs6590343 | *FLI1* | -0.012 (-0.016, -0.008) | 6.5e-09 | Yes | Yes | 0.005 (-0.058, 0.068) | 0.869 |
| 16 | rs12447324 | *PAPD5* | -0.015 (-0.021, -0.010) | 1.1e-08 | Yes | Yes | 0.057 (-0.031, 0.144) | 0.202 |
| 12 | rs1907702 | *KITLG* | -0.014 (-0.019, -0.009) | 1.2e-08 | Yes | Yes | 0.015 (-0.062, 0.092) | 0.701 |
| 18 | rs78694226 | *TYMS* | 0.066 (0.043, 0.089) | 1.6e-08 | Yes | Yes | 0.016 (-0.292, 0.324) | 0.919 |
| 17 | rs34405642 | *NOL11* | -0.012 (-0.017, -0.008) | 2.4e-08 | Yes | Yes | -0.012 (-0.082, 0.058) | 0.729 |
| 19 | rs11084431 | *ZSCAN5B* | 0.012 (0.008, 0.016) | 2.6e-08 | Yes | No | - | - |
| 16 | rs450962 | *EIF3CL* | -0.014 (-0.019, -0.009) | 2.9e-08 | Yes | No | - | - |
| 18 | rs6565924 | *ZNF236* | -0.012 (-0.016, -0.008) | 4.1e-08 | Yes | Yes | 0.025 (-0.038, 0.089) | 0.435 |
| 8 | rs7012816 | *PRDM14* | -0.017 (-0.023, -0.011) | 4.4e-08 | Yes | Yes | -0.063 (-0.153, 0.026) | 0.167 |
| 20 | rs55765053 | *RTEL1* | -0.022 (-0.030, -0.014) | 5.1e-08 | No | Yes | -0.106 (-0.253, 0.041) | 0.157 |
| 2 | rs35671754 | *ATIC* | -0.012 (-0.017, -0.008) | 5.9e-08 | Yes | Yes | -0.038 (-0.103, 0.028) | 0.263 |
| 11 | rs334 | *HBB* | -0.665 (-0.989, -0.342)* | 5.6e-05 | No | Yes | - | - |
| 11 | rs1609812 | *HBB* | -0.046 (-0.052, -0.041) | 6.3e-59 | No | Yes | 0.070 (-0.01, 0.15) | 0.085 |

1. **Prior GWAS of measured leukocyte telomere length by Li et. al 2021**

| **Prior GWAS** | | | | | | | **Replication in**  **the Rhineland Study** | | |
| --- | --- | --- | --- | --- | --- | --- | --- | --- | --- |
| **Chr** | **SNP** | **Gene** | **EA** | **Estimate** | **SE** | **P** | **Estimate** | **SE** | **P** |
| **Genome-wide significance (5*10^-8^)** | | | | | | | | | |
| ***3*** | ***rs10936600*** | ***LRRC34 (TERC)*** | ***T*** | ***-0.0858*** | ***0.0057*** | ***6.42E-51*** | ***-0.1098*** | ***0.0354*** | ***0.0020*** |
| 5 | rs7705526 | *TERT* | A | 0.0820 | 0.0058 | 4.82E-45 | 0.0394 | 0.0375 | 0.2946 |
| ***5*** | ***rs2853677*** | ***TERT*** | ***A*** | ***-0.0638*** | ***0.0055*** | ***3.12E-31*** | ***-0.0705*** | ***0.0321*** | ***0.0282*** |
| 4 | rs4691895 | *NAF1* | C | 0.0577 | 0.0061 | 1.47E-21 | 0.0266 | 0.0370 | 0.4727 |
| ***10*** | ***rs9419958*** | ***STN1 (OBFC1)*** | ***C*** | ***-0.0636*** | ***0.0071*** | ***4.77E-19*** | ***-0.0814*** | ***0.0446*** | ***0.0681*** |
| ***20*** | ***rs75691080*** | ***STMN3*** | ***T*** | ***-0.0671*** | ***0.0089*** | ***5.75E-14*** | ***-0.1578*** | ***0.0559*** | ***0.0048*** |
| 7 | rs59294613 | *POT1* | A | -0.0407 | 0.0055 | 1.12E-13 | -0.0108 | 0.0342 | 0.7512 |
| 19 | rs8105767 | *ZNF208* | G | 0.0392 | 0.0054 | 5.21E-13 | 0.0557 | 0.0342 | 0.1035 |
| 20 | rs73624724 | *ZBTB46* | C | 0.0507 | 0.0074 | 6.08E-12 | -0.0088 | 0.0477 | 0.8533 |
| 1 | rs3219104 | *PARP1* | C | 0.0417 | 0.0064 | 9.31E-11 | 0.0128 | 0.0406 | 0.7519 |
| 20 | rs932827 | *ZBTB46* | T | -0.0374 | 0.0060 | 3.28E-10 | -0.0557 | 0.0358 | 0.1200 |
| 6 | rs2736176 | *PRRC2A* | C | 0.0345 | 0.0055 | 3.41E-10 | 0.0345 | 0.0360 | 0.3378 |
| 16 | rs3785074 | *TERF2* | G | 0.0351 | 0.0056 | 4.50E-10 | 0.0259 | 0.0357 | 0.4684 |
| 16 | rs7194734 | *MPHOSPH6* | T | -0.0369 | 0.0060 | 6.72E-10 | -0.0032 | 0.0388 | 0.9339 |
| 20 | rs34978822 | *RTEL1* | G | -0.1397 | 0.0227 | 7.04E-10 | 0.0303 | 0.1284 | 0.8137 |
| 6 | rs34991172 | *CARMIL1* | G | -0.0608 | 0.0105 | 6.03E-09 | -0.0929 | 0.0739 | 0.2089 |
| 11 | rs228595 | *ATM* | A | -0.0285 | 0.0050 | 1.39E-08 | 0.0030 | 0.0317 | 0.9247 |
| 14 | rs2302588 | *DCAF4* | C | 0.0476 | 0.0084 | 1.64E-08 | -0.0217 | 0.0503 | 0.6664 |
| 4 | rs13137667 | *MOB1B* | C | 0.0765 | 0.0137 | 2.37E-08 | 0.0383 | 0.0906 | 0.6723 |
| 3 | rs55749605 | *SENP7* | A | -0.0373 | 0.0067 | 2.38E-08 | 0.0342 | 0.0324 | 0.2902 |
| 16 | rs62053580 | *RFWD3* | G | -0.0389 | 0.0071 | 3.96E-08 | -0.0414 | 0.0413 | 0.3157 |
| **False-discovery rate < 0.05** | | | | | | | | | |
| 2 | rs754017156 | *ACYP2* | D | 0.0471 | 0.0088 | 7.52E-08 | - | - | - |
| 15 | rs12909131 | *ATP8B4* | T | -0.0308 | 0.0058 | 1.15E-07 | 0.0132 | 0.0363 | 0.7150 |
| 20 | rs1744757 | *MROH8* | T | 0.0359 | 0.0068 | 1.38E-07 | 0.0441 | 0.0454 | 0.3314 |
| 18 | rs2124616 | *TYMS* | A | -0.0374 | 0.0072 | 1.72E-07 | -0.0232 | 0.0433 | 0.5930 |
| 3 | rs2613954 | *RP11-572M11.4* | T | -0.0381 | 0.0078 | 1.10E-06 | 0.0074 | 0.0473 | 0.8766 |
| 1 | rs12065882 | *MAGI3* | G | 0.0298 | 0.0062 | 1.36E-06 | 0.0078 | 0.0377 | 0.8367 |
| 10 | rs2386642 | *ASB13* | A | -0.0256 | 0.0053 | 1.44E-06 | -0.0112 | 0.0327 | 0.7312 |
| 2 | rs56810761 | *UNC80* | T | 0.0275 | 0.0057 | 1.45E-06 | 0.0165 | 0.0352 | 0.6392 |
| 5 | rs62365174 | *TENT2* | G | -0.0544 | 0.0113 | 1.50E-06 | 0.0486 | 0.0530 | 0.3591 |
| 12 | rs112655343 | *ATF7IP* | T | 0.0425 | 0.0090 | 2.22E-06 | 0.0826 | 0.0514 | 0.1087 |
| 15 | rs55710439 | *ANKDD1A* | T | 0.1050 | 0.0223 | 2.65E-06 | 0.0891 | 0.1378 | 0.5180 |
| 16 | rs11640926 | *CACNA1H* | G | 0.0557 | 0.0119 | 2.93E-06 | -0.0538 | 0.0461 | 0.2427 |
| 4 | rs60160057 | *DCLK2* | A | -0.0287 | 0.0062 | 3.15E-06 | 0.0248 | 0.0381 | 0.5158 |
| 14 | rs117536281 | *CDCA4* | G | 0.0850 | 0.0183 | 3.31E-06 | -0.0471 | 0.0977 | 0.6298 |
| 22 | rs7510583 | *KIAA1644* | G | 0.0347 | 0.0075 | 3.38E-06 | - | - | - |
| 14 | rs59192843 | *BBOF1 (CCDC176)* | G | 0.0655 | 0.0141 | 3.52E-06 | 0.0310 | 0.0748 | 0.6783 |
| ***8*** | ***rs57415150*** | ***CSMD1*** | ***A*** | ***-0.0584*** | ***0.0126*** | ***3.68E-06*** | ***-0.1557*** | ***0.0741*** | ***0.0357*** |
| 20 | rs6038821 | *LINC01706* | T | 0.0596 | 0.0129 | 3.98E-06 | -0.0044 | 0.0835 | 0.9584 |
| 17 | rs144204502 | *TK1* | T | -0.0896 | 0.0196 | 4.92E-06 | -0.0359 | 0.1300 | 0.7824 |
| 20 | rs6107615 | *PROKR2* | C | -0.0228 | 0.0050 | 5.30E-06 | 0.0040 | 0.0315 | 0.9002 |
| 15 | rs9972513 | *RP11-275I4.2* | T | 0.0247 | 0.0055 | 5.75E-06 | - | - | - |
| 11 | rs117037102 | *CEP295* | T | 0.0979 | 0.0218 | 6.81E-06 | -0.0469 | 0.1535 | 0.7600 |
| 21 | rs7276273 | *KRTAP10-4* | C | -0.1502 | 0.0334 | 6.90E-06 | -0.3428 | 0.2033 | 0.0920 |
| 19 | rs11665818 | *IFNL2* | A | 0.0278 | 0.0062 | 7.04E-06 | 0.0012 | 0.0407 | 0.9765 |
| 14 | rs3213718 | *CALM1* | T | 0.0224 | 0.0050 | 7.22E-06 | 0.0195 | 0.0323 | 0.5458 |
| 5 | rs112347796 | *UBE2D2* | D | 0.0691 | 0.0154 | 7.29E-06 | - | - | - |
| 19 | rs143276018 | *NMRK2* | C | -0.1015 | 0.0229 | 9.02E-06 | 0.0020 | 0.1411 | 0.9887 |
| 8 | rs201375979 | *COX6C* | D | 0.0332 | 0.0075 | 9.11E-06 | - | - | - |
| 12 | rs7311314 | *SMUG1* | A | 0.0240 | 0.0054 | 9.50E-06 | 0.0069 | 0.0334 | 0.8362 |
| 1 | rs35675808 | *CD247* | G | 0.0736 | 0.0166 | 9.54E-06 | -0.0712 | 0.1106 | 0.5201 |
| 15 | rs117610974 | *UNC13C* | G | -0.1540 | 0.0350 | 1.05E-05 | 0.0887 | 0.1578 | 0.5740 |

Estimate: the per-allele effect on z-scored LTL. Abbreviation: GWAS, genome-wide association study; Chr, chromosome; SNP, single nucleotide polymorphism; EA, effect allele; SE, standard error; MR, Mendelian randomization.

**Table S2. Gene ontology enrichment analyses of nearest genes associated with leukocyte telomere length measurements**

| **Trait** | **Term ID** | **Name** | **Log_10_(p-value)** | **Log_10_(size)** | **Frequency** | **Uniqueness** | **Dispensability** |
| --- | --- | --- | --- | --- | --- | --- | --- |
| **Measured LTL** | |  |  |  |  |  |  |
|  | GO:0052547 | regulation of peptidase activity | -3.29 | 2.66 | 2.53 | 1.00 | 0 |
| **Genetically predicted LTL** | | | |  |  |  |  |
|  | GO:0097035 | regulation of membrane lipid distribution | -3.57 | 1.81 | 0.35 | 1.00 | 0 |
| **Delta LTL** |  |  |  |  |  |  |  |
|  | GO:0030111 | regulation of Wnt signaling pathway | -5.17 | 2.51 | 1.82 | 0.81 | 0 |
|  | GO:0060828 | regulation of canonical Wnt signaling pathway | -3.85 | 2.4 | 1.39 | 0.77 | 0.36 |
|  | GO:0001764 | neuron migration | -3.74 | 2.15 | 0.78 | 0.96 | 0 |
|  | GO:0090314 | positive regulation of protein targeting to membrane | -3.71 | 1.48 | 0.16 | 0.85 | 0.03 |
|  | GO:1905114 | cell surface receptor signaling pathway involved in cell-cell signaling | -3.7 | 2.6 | 2.22 | 0.85 | 0.04 |
|  | GO:0009451 | RNA modification | -3.68 | 2.25 | 1.00 | 1.00 | 0.01 |
|  | GO:0016043 | cellular component organization | -3.23 | 3.74 | 30.57 | 0.99 | 0.01 |
|  | GO:0071840 | cellular component organization or biogenesis | -3.22 | 3.75 | 31.7 | 0.99 | 0.02 |
|  | GO:0021814 | cell motility involved in cerebral cortex radial glia guided migration | -3.14 | 0.90 | 0.04 | 0.96 | 0.45 |
|  | GO:0030948 | negative regulation of vascular endothelial growth factor receptor signaling pathway | -3.14 | 1.08 | 0.06 | 0.81 | 0.33 |
|  | GO:0034144 | negative regulation of toll-like receptor 4 signaling pathway | -3.14 | 1.04 | 0.06 | 0.81 | 0.26 |
|  | GO:0035924 | cellular response to vascular endothelial growth factor stimulus | -3.14 | 1.64 | 0.24 | 0.96 | 0.13 |
|  | GO:0036324 | vascular endothelial growth factor receptor-2 signaling pathway | -3.14 | 0.78 | 0.03 | 0.90 | 0.31 |
|  | GO:0038026 | reelin-mediated signaling pathway | -3.14 | 0.95 | 0.04 | 0.9 | 0.32 |
|  | GO:0090129 | positive regulation of synapse maturation | -3.14 | 1.00 | 0.05 | 0.93 | 0.11 |
|  | GO:1903726 | negative regulation of phospholipid metabolic process | -3.14 | 0.9 | 0.04 | 0.85 | 0.03 |
|  | GO:1905898 | positive regulation of response to endoplasmic reticulum stress | -3.14 | 1.56 | 0.20 | 0.71 | 0.23 |
|  | GO:2001224 | positive regulation of neuron migration | -3.14 | 1.28 | 0.10 | 0.9 | 0.24 |
|  | GO:0042753 | positive regulation of circadian rhythm | -3.05 | 1.28 | 0.10 | 0.93 | 0.10 |

Log_10_(p-value): base-10 logarithm of the p-value for over-representation of the gene set. Log_10_(size): base-10 logarithm of the number of annotations for GO term ID in the EBI GOA database. Uniqueness measures whether the term is an outlier when compared semantically to the whole list (regardless of the p-values). Dispensability compares a term to other semantically close terms and is assigned based on both the semantic distance and the supplied p-values.

**Table S3. KEGG pathway analyses of nearest genes associated with leukocyte telomere length measurements**

| **Trait** | **Term ID** | **Description** | **N** | **DE** | **P-value** | **Significant genes in gene set** |
| --- | --- | --- | --- | --- | --- | --- |
| **Measured LTL** | | | | | | |
|  | path:hsa05220 | Chronic myeloid leukemia | 74 | 5 | 9.58e-03 | *CRK,AKT2,ABL1,PTPN11,SOS1* |
|  | path:hsa04936 | Alcoholic liver disease | 139 | 6 | 9.60e-03 | *CYP2E1,AKT2,LPIN1,PRKAG3,MAP3K7,TRAF3* |
|  | path:hsa04722 | Neurotrophin signaling pathway | 114 | 6 | 1.37e-02 | *CRK,AKT2,ABL1,NTRK3,PTPN11,SOS1* |
|  | path:hsa00310 | Lysine degradation | 61 | 4 | 1.73e-02 | *PLOD1,SETMAR,SMYD3,PRDM2* |
|  | path:hsa05211 | Renal cell carcinoma | 65 | 4 | 2.54e-02 | *CRK,AKT2,PTPN11,SOS1* |
|  | path:hsa04620 | Toll-like receptor signaling pathway | 100 | 4 | 2.76e-02 | *AKT2,TOLLIP,MAP3K7,TRAF3* |
|  | path:hsa05171 | Coronavirus disease - COVID-19 | 218 | 6 | 3.01e-02 | *ACE,RPL27A,RPL41,RPS13,MAP3K7,TRAF3* |
|  | path:hsa05168 | Herpes simplex virus 1 infection | 477 | 10 | 4.08e-02 | *ZNF792,AKT2,PML,PTPN11,MAP3K7,TAP2,TRAF3,ZNF12,ZNF224,ZNF514* |
|  | path:hsa05208 | Chemical carcinogenesis - reactive oxygen species | 202 | 6 | 4.63e-02 | *CYP2E1,AKT2,ABL1,PTPN11,SOS1,UQCRC1* |
|  | path:hsa04120 | Ubiquitin mediated proteolysis | 134 | 5 | 5.11e-02 | *PRKN,PML,BIRC6,HERC1,UBE3C* |
| **Genetically predicted LTL** | | | | | | |
|  | path:hsa00591 | Linoleic acid metabolism | 30 | 4 | 2.35e-02 | *CYP1A2,CYP2C19,PLA2G4D,PLA2G2C* |
|  | path:hsa05160 | Hepatitis C | 153 | 13 | 3.22e-02 | *AKT3,CDK4,CLDN7,TICAM1,EGFR,GRB2,PPP2R1B,PPP2R2C,PPP2R2D,SOS2,STAT2,TRAF2,YWHAB,CLDN10* |
|  | path:hsa04926 | Relaxin signaling pathway | 126 | 12 | 5.27e-02 | *AKT3,ADCY2,COL1A1,EDNRB,EGFR,GNG5,GNG7,GRB2,SMAD2,ARRB2,PRKACG,SOS2,VEGFB* |
|  | path:hsa03060 | Protein export | 23 | 3 | 6.59e-02 | *SEC61G,SRP19,IMMP2L* |
|  | path:hsa04072 | Phospholipase D signaling pathway | 144 | 14 | 6.73e-02 | *AKT3,AGPAT1,ADCY2,PIK3R6,EGFR,GNA12,PLA2G4D,GRB2,GRM2,GRM4,GRM5,PDGFRA,SOS2,DGKZ,DGKD* |
|  | path:hsa00410 | beta-Alanine metabolism | 31 | 4 | 6.87e-02 | *ALDH3B2,ACADS,CNDP1,AOC3* |
|  | path:hsa04136 | Autophagy - other | 30 | 4 | 6.92e-02 | *ATG7,WIPI1,RPTOR,ATG10,ATG4D* |
|  | path:hsa04540 | Gap junction | 87 | 9 | 7.09e-02 | *ADCY2,EGFR,GRB2,GRM5,GUCY1A1,PDGFRA,PRKACG,SOS2,CDK1* |
|  | path:hsa00520 | Amino sugar and nucleotide sugar metabolism | 48 | 5 | 7.99e-02 | *GALK1,GFPT1,CYB5R1,UAP1,UXS1* |
|  | path:hsa04115 | p53 signaling pathway | 73 | 7 | 8.46e-02 | *CDK4,BCL2,RRM2,TNFRSF10B,CCND3,GORAB,CDK1* |
| **Delta LTL** | | | | | | |
|  | path:hsa00601 | Glycosphingolipid biosynthesis - lacto and neolacto series | 27 | 6 | 1.77e-02 | *B3GNT3,ABO,A4GALT,ST3GAL4,ST3GAL3,B3GALNT1* |
|  | path:hsa04512 | ECM-receptor interaction | 86 | 17 | 2.40e-02 | *LAMC3,COL4A1,COL4A2,COL6A3,COL9A1,SV2C,TNC,AGRN,LAMA4,LAMC1,SDC1,THBS4,TNXB,VWF,FRAS1,CD44,SV2B* |
|  | path:hsa04928 | Parathyroid hormone synthesis, secretion and action | 105 | 20 | 2.95e-02 | *ADCY2,ADCY5,ATF6B,EGFR,FGFR1,GNA12,GNAQ,ITPR1,LRP5,ARRB1,MEF2C,MMP16,MMP17,PDE4C,PDE4D,PRKCA,MAPK1,MAP2K1,RXRB,SLC34A1,SLC9A3R1* |
|  | path:hsa04520 | Adherens junction | 70 | 15 | 3.54e-02 | *SORBS1,CTNNB1,EGFR,FGFR1,FYN,CTNNA3,IGF1R,LMO7,MAPK1,PTPRB,PTPRF,TCF7L2,VCL,TCF7L1,CDC42* |
|  | path:hsa04929 | GnRH secretion | 62 | 13 | 4.13e-02 | *ESR2,GABBR1,GNAQ,HCN1,ITPR1,KCNJ6,KCNN1,KCNN3,ARRB1,PRKCA,MAPK1,MAP2K1,CACNA1C,CACNA1D* |
|  | path:hsa04061 | Viral protein interaction with cytokine and cytokine receptor | 97 | 9 | 4.79e-02 | *CCL26,CSF1,CX3CR1,IL2RA,CXCR1,IL20RB,CX3CL1,CCR2,IL18R1* |
|  | path:hsa05217 | Basal cell carcinoma | 63 | 11 | 5.05e-02 | *CTNNB1,GLI2,GLI3,SUFU,TCF7L2,WNT5A,WNT8B,FZD5,FZD3,FZD6,TCF7L1* |
|  | path:hsa00514 | Other types of O-glycan biosynthesis | 45 | 9 | 5.27e-02 | *GXYLT1,EOGT,GALNT18,LFNG,GALNTL6,GALNT9,POGLUT1,ST6GAL1,ST3GAL3* |
|  | path:hsa04270 | Vascular smooth muscle contraction | 132 | 20 | 5.58e-02 | *ADCY2,ADCY5,PLA2G4E,GNA12,GNAQ,PLA2G4D,ITPR1,KCNMA1,MYH11,MYLK,PPP1R12A,PRKCA,PRKCH,MAPK1,MAP2K1,PTGIR,CACNA1C,CACNA1D,CALCA,PLA2G6* |
|  | path:hsa04934 | Cushing syndrome | 154 | 24 | 6.30e-02 | *ADCY2,ADCY5,ATF6B,CRHR2,CTNNB1,EGFR,GNAQ,ITPR1,KMT2A,PDE8A,MAPK1,MAP2K1,TCF7L2,WNT5A,WNT8B,CACNA1C,CACNA1D,FZD5,FZD3,ARMC5,FZD6,TCF7L1,ORAI1,ASH2L* |

N: number of genes in the gene set. DE: number of genes that are differentially methylated. P-value, p-value for over-representation of the gene set**.**
